# Closing the life-cycle of normative modeling using federated hierarchical Bayesian regression

**DOI:** 10.1371/journal.pone.0278776

**Published:** 2022-12-08

**Authors:** Seyed Mostafa Kia, Hester Huijsdens, Saige Rutherford, Augustijn de Boer, Richard Dinga, Thomas Wolfers, Pierre Berthet, Maarten Mennes, Ole A. Andreassen, Lars T. Westlye, Christian F. Beckmann, Andre F. Marquand

**Affiliations:** 1 Donders Institute for Brain, Cognition and Behaviour, Radboud University, Nijmegen, The Netherlands; 2 Department for Cognitive Neuroscience, Radboud University Medical Center, Nijmegen, The Netherlands; 3 Department of Psychiatry, University Medical Center Utrecht, Utrecht, The Netherlands; 4 Department of Psychology, University of Oslo, Oslo, Norway; 5 Norwegian Centre for Mental Disorders Research, Institute of Clinical Medicine, University of Oslo, Oslo, Norway; 6 Division of Mental Health and Addiction, Oslo University Hospital, Oslo, Norway; 7 Centre for Functional MRI of the Brain, University of Oxford, Oxford, United Kingdom; 8 Department of Neuroimaging, Institute of Psychiatry, King’s College London, London, United Kingdom; University of North Carolina at Chapel Hill, UNITED STATES

## Abstract

Clinical neuroimaging data availability has grown substantially in the last decade, providing the potential for studying heterogeneity in clinical cohorts on a previously unprecedented scale. Normative modeling is an emerging statistical tool for dissecting heterogeneity in complex brain disorders. However, its application remains technically challenging due to medical data privacy issues and difficulties in dealing with nuisance variation, such as the variability in the image acquisition process. Here, we approach the problem of estimating a *reference* normative model across a massive population using a massive multi-center neuroimaging dataset. To this end, we introduce a federated probabilistic framework using hierarchical Bayesian regression (HBR) to complete the life-cycle of normative modeling. The proposed model provides the possibilities to learn, update, and adapt the model parameters on decentralized neuroimaging data. Our experimental results confirm the superiority of HBR in deriving more accurate normative ranges on large multi-site neuroimaging datasets compared to the current standard methods. In addition, our approach provides the possibility to recalibrate and reuse the learned model on local datasets and even on datasets with very small sample sizes. The proposed method will facilitate applications of normative modeling as a medical tool for screening the biological deviations in individuals affected by complex illnesses such as mental disorders.

## Introduction

*Normative modeling* was recently introduced as a statistical framework for studying the biological heterogeneity of mental disorders in clinical neuroimaging cohorts [1]. Normative modeling involves estimating the centiles of variation, *i.e*., the normative ranges, of a biological brain measure (*e.g*., ROI cortical thickness, ROI volume, functional connectivity) as a function of clinical covariates. This is performed via regressing the units of neuroimaging data (*e.g*., a voxel in structural or functional MRIs) against a set of clinically relevant covariates (*e.g*., age). Analogous to the use of ‘growth charts’ in pediatric medicine, such a mapping function provides a norm for the changes in the structure or functional dynamics of the brain across the human lifespan [2]. Deviations of individuals from the normative range can be quantified as z-scores [3]. This approach has recently been used to dissect the heterogeneity of several mental disorders [4–7], providing compelling evidence that brain abnormalities of patients with psychiatric disorders cannot be captured in a case-control setting, *i.e*., by average group differences between patients with a specific disorder and healthy controls. Thus, normative modeling allows us to enhance classical symptom-based diagnostics by incorporating biological and environmental factors in a principled way. Such a paradigm change will hopefully result in developing effective biological tests and individualized treatments to improve the quality of life of patients with psychiatric, neurodevelopmental, and neurodegenerative disorders [8, 9].

The success of normative modeling depends on the accuracy of estimating the norm and the variability around this norm for a certain brain measure (or putative biomarker) across a population. Therefore, massive data availability from a large and diverse population, extensive computational resources, and intelligent modeling techniques play pivotal roles. The advancements in data sharing standards [10] and protocols [11–13] led to an exponential growth in neuroimaging data availability. Neuroimaging groups worldwide join forces in international consortia leading to clinical neuroimaging studies that are orders of magnitude larger today than a decade ago [10, 14, 15]. This trend has just begun, and with the recent advances in high-performance computing technologies such as grid computing, cloud computing, and GPU technologies, we now possess enough computational power to store and process these massive datasets. Furthermore, progress in artificial intelligence and machine learning over the last decades brought ubiquitous applications in healthcare. These developments are the foundations for large-scale normative modeling.

In this article, we attack the problem of estimating a *reference* normative model on decentralized multi-center neuroimaging data. Developing such a reference normative model is challenged in practice by two main obstacles. First, it requires aggregating smaller neuroimaging datasets acquired at several imaging centers with different acquisition protocols and scanners. Furthermore, the data is often processed using various preprocessing pipelines and toolboxes, each of which leaves its signature on the final derived statistics [16], referred to as *site-effects*. Site-effects introduce artefactual variability in data which confounds the derived deviations in normative modeling [3]. Thus, the practical application of normative modeling as a medical tool is limited as the data collected at different centers may express variable characteristics. To this end, developing effective methods to deal with these confounds is essential. The second barrier in deriving a reference normative model and deploying it as a medical tool at local clinical centers is data privacy [17, 18]. Clinical data are always subject to privacy regulations and cannot be distributed freely without acquiring appropriate consent. This fact challenges the centralized model estimation in which the model estimation algorithm requires access to whole data at once. Therefore, it is essential to decentralize the model estimation phase by developing a federated learning [18–21] approach for normative modeling.

Federated learning (FL) [19] offers a natural solution for decentralizing the learning process of a reference normative model on distributed data. In FL, multiple local data centers (clients) collaborate in learning the parameters of a machine learning model [22]. This process is generally orchestrated by a central server that handles the distribution and aggregation of model parameters. In this scheme, the data are stored locally and are not transferred across data centers during the model estimation process. Therefore, FL addresses the data sharing and privacy issues in applications of machine learning in the medical domains [18]. However, applying FL methods in practice poses several algorithmic and practical challenges such as computational and communication complexities, non-IID (independently and identically distributed) data, and unbalanced sample distribution [22]. The latter two problems are ubiquitous in the neuroimaging context as data collected across different centers are generally non-IID (due to the site-effect) and unbalanced (some are with big and some are with very small sample size).

In this study, we first sketch the life-cycle of normative modeling. Then, we show how a hierarchical Bayesian regression (HBR) [23] model can be employed to close this life-cycle. To this end, we extend our previous effort in using HBR for multi-site normative modeling [24] by introducing a fully probabilistic federated learning framework for normative modeling on decentralized neuroimaging data. Our method offers several notable features: i) it provides the possibility of federated model estimation/calibration on decentralized data; ii) it can handle the site-effect in multi-center neuroimaging data without the need for data harmonization, thus largely avoids its shortcomings (*e.g*., removing too much variance when site effects are correlated with effects of interest); iii) it is suitable for federated learning on non-IID data from multiple sites; iv) it offers a mechanism for few-shot learning on small local data thus is suitable for federated learning on unbalanced data distributions; v) given its probabilistic nature, it provides estimations of phenomenological variability in data and epistemological uncertainty in the model [25], thus is well-suited for normative modeling; and vi) it is highly flexible and accommodates different modeling choices (*e.g*., non-linear effects or heteroscedastic noise). More specifically, our contribution extends our previous conference publication [24] in methodological and experimental aspects. From the methodological point of view, here and for the first time, we use the generative nature of the HBR model to estimate/update model parameters in a federative manner and on decentralized data. From the experimental point of view, we scaled up the size of our experimental data from 7 to 16 datasets, from 33 to 79 scanners, and from 7499 to 37126 scans. We have also added several experiments including i) performance comparison with polynomial and B-spline models, ii) the performance comparison between centralized and decentralized scenarios, and iii) few-shot learning in the extremely unbalanced data distribution. Our experimental results on massive neuroimaging data demonstrate the effectiveness of the proposed FL framework in several scenarios for developing and deploying a reference normative model on decentralized data.

## Materials and methods

In this section, we discuss the components involved in this life-cycle of normative modeling and their technical requirements in model development and deployment stages. After formalizing the definition of normative modeling in a machine learning setting, we review possible existing solutions for normative modeling on multi-site data and their limitations. Then, we show how the hierarchical Bayesian framework is used to overcome these limitations and close the life-cycle of normative modeling. Finally, we describe the experimental materials and setups used to validate the proposed solution.

### Normative modeling: The life-cycle

**Fig 1:**
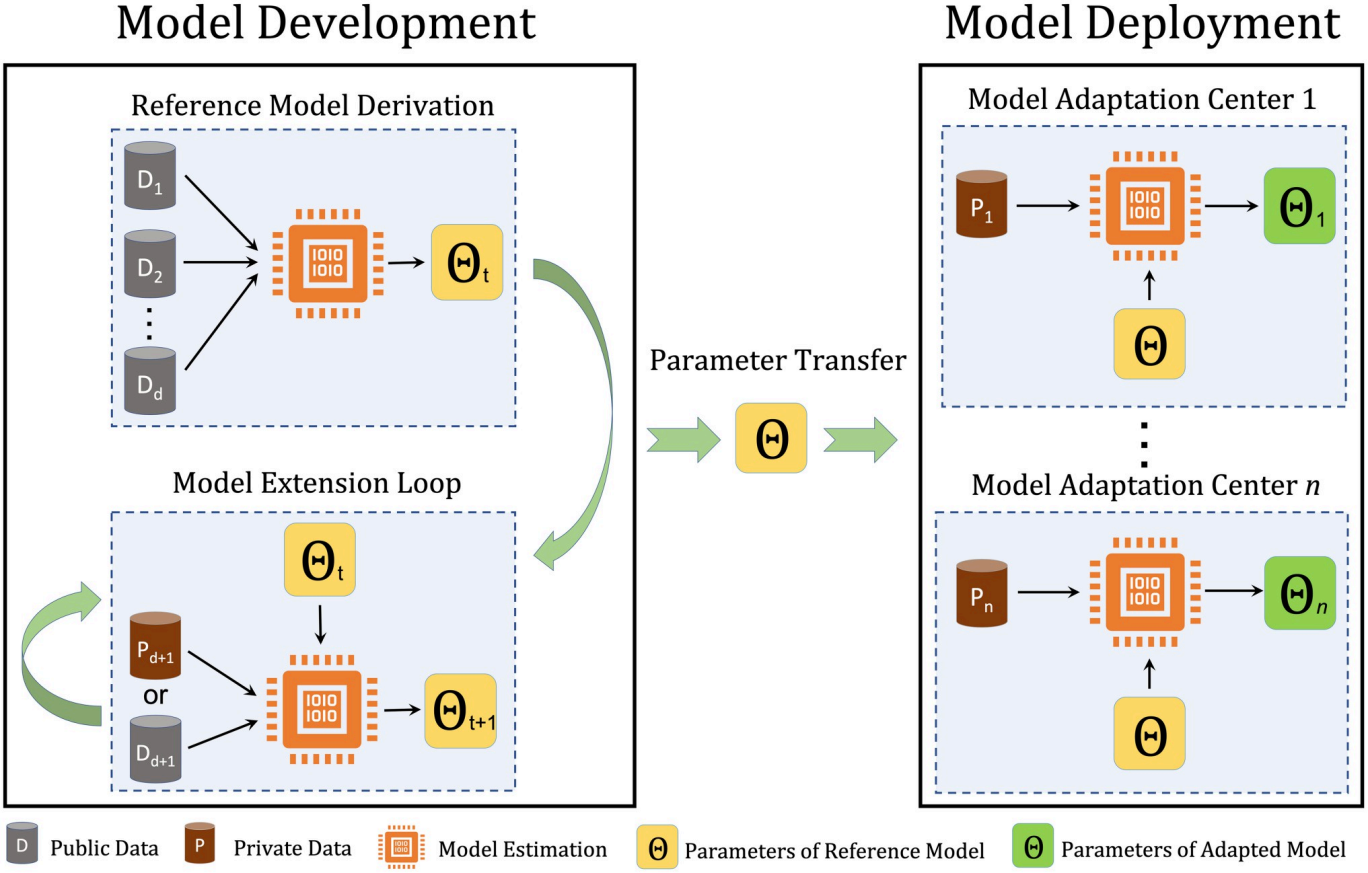
Model development and model deployment in the normative model life-cycle. In the model development phase, the parameters of the reference model are estimated on *d* datasets (*D*_1_, *D*_2_, …, *D*_*d*_). The model extension loop provides the possibility of model development on decentralized data at time point *t*. In the model deployment phase, the parameters of the reference model are adapted to local data at hospitals or research centers.

The complete pipeline of normative modeling, Fig 1, is comprised of two main components i) model development and ii) model deployment. The model development refers to the i) estimation of an early version of a reference model on a multi-center initial dataset and ii) iterative and cyclic process of updating its parameters on newly observed data from new centers over time. The model deployment refers to the process of adapting the parameters of the reference model to local data, *e.g*., at local hospitals or research centers. We refer to this complete pipeline as a *life-cycle* because it contains all the operations needed for estimating, updating, adapting, and applying a reference normative model.

However, implementing the life-cycle of normative modeling is not straightforward due to the real-world limitations in multi-site data analysis and data privacy/access issues. To address these issues, the modeling approach that is employed for estimating the parameters of the normative model must have four vital features:

1. It should be able to deal with site-effects;
2. In the development phase, it should have the possibility of updating the parameters of the reference model over time and when new datasets are available, without requiring access to the full primary dataset. We refer to this process as model extension;
3. It should apply to both centralized and decentralized data. The centralized data refers to the scenario in which all training data are available for model estimation. In the decentralized case, the data are distributed across different centers, and data sharing and transfer are not possible due to privacy issues;
4. In the deployment phase, it should provide a mechanism for adapting the parameters of the reference model to novel data at the deployment centers (*e.g*., local hospitals). It is crucial to emphasize that the initial data used for estimating the reference model might not be available during the adaptation process. Therefore, the knowledge transform must be performed using a parameter transfer learning approach [26]. We refer to this process as model adaptation.

In the remaining text of this section, we present practical solutions for implementing these features. To this end, we first formally define the normative modeling procedure.

### Normative modeling: The formal definition

Let $\text{X}\in R^{n\times p}$ represent a matrix of *p* clinical covariates for *n* participants. We denote the corresponding neuroimaging measures at each measurement unit (*e.g*., a voxel) by $\text{y}\in R^{n}$. Assuming a Gaussian distribution over each neuroimaging measure, *i.e*., $y∼N(\mu ,\sigma^{2})$, in normative modeling we are interested in finding a parametric or non-parametric form for *μ* and *σ* given the covariates in **X**. Then, for example, *μ* ± 1.96*σ* forms the 95% percentile for the normative range of **y**. To estimate *μ* and *σ*, we parametrize them respectively on *f*_*μ*_(**X**, *θ*_*μ*_) and $f_{\sigma }^{+}\left(\text{X},\theta_{\sigma }\right)$, where *θ*_*μ*_ and *θ*_*σ*_ are the parameters of *f*_*μ*_ and $f_{\sigma }^{+}$. Here, $f_{\sigma }^{+}$ is a non-negative function that estimates the standard deviation of heteroscedastic noise in data. The homoscedastic formulation is a specific case where *σ* is independent of **X**. The non-negativity of $f_{\sigma }^{+}$ can be enforced for example using the *softplus* function $f_{\sigma }^{+}=log\left(1+\text{exp}\left(f_{\sigma }\right)\right)$ [27–29].

In the normative modeling context, the deviations of samples from the normative range are quantified as z-scores [1]:

$$\begin{array}{c} \text{z}=\frac{\text{y}-f_{\mu }\left(\text{X},\theta_{\mu }\right)}{f_{\sigma }^{+}\left(\text{X},\theta_{\sigma }\right)}. \end{array}$$ (1)

As discussed, to close the application loop for normative modeling, the model must accommodate multi-site data. We discuss classical strategies for multi-site neuroimaging data modeling in the next section.

### Multi-site normative modeling

Let ${\text{y}}_{i}\in R^{n_{i}}$ denote neuroimaging measures for *n*_*i*_ participants in the *i*th group, *i* ∈ {1, …, *m*}, of data and we have $y_{i}∼N\left(\mu_{i},\sigma_{i}^{2}\right)$. Here, a group refers to any non-ordinal categorical variable such as a batch-effect (that causes unwanted and non-biological variation in data) or other biologically relevant variables such as sex or ethnicity. In this article, since the focus is on multi-site normative modeling, we use the term ‘batch’ to refer to each group (otherwise mentioned) where each batch refers to data that are collected at different imaging sites. However, our formulations are general for application on other possible batch-effects (*e.g*., processing software version) or biologically relevant group-effects (*e.g*., sex and ethnicity).

Traditionally, there are four possible strategies for normative modeling on multi-site data. In the following, we explain the theoretical and practical limitations of these approaches in the life-cycle of normative modeling.

#### Naive pooling

**Fig 2:**
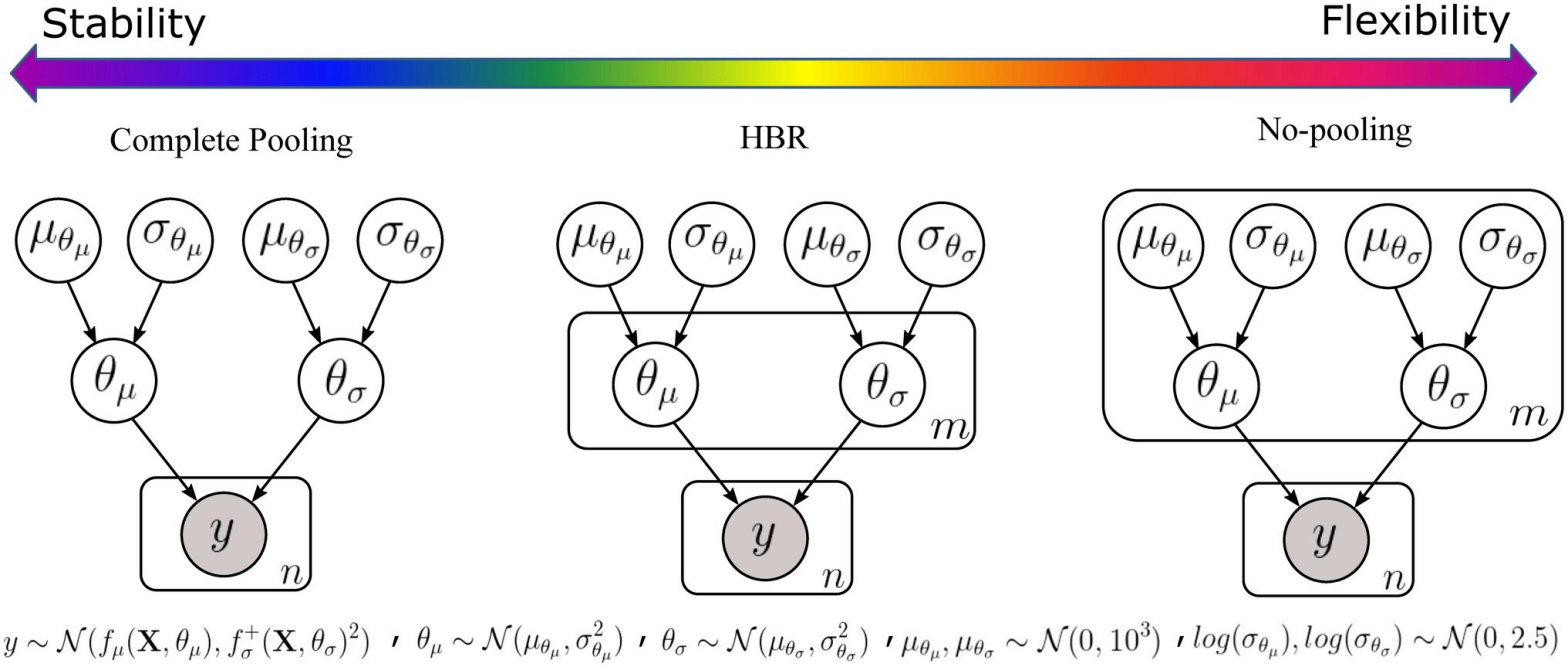
Graphical models of complete pooling, partial pooling via HBR, and no-pooling. The solutions for handling the site-effects form a spectrum in the model stability-flexibility space. At the stability end of the spectrum, we have the complete pooling solution. In the complete pooling scenario, the model learns the same set of parameters and hyperparameters on big data. At the flexibility end of the spectrum stands the no-pooling approach, where a large set of parameters and hyperparameters are estimated for each site, however, it does not benefit from the richness of big data. Therefore, its parameters can be unstable for sites with small sample size. The HBR lies in the middle of the spectrum, thus, it brings the best of two worlds together. In HBR, similar to no-pooling, we allow the model to learn different sets of parameters for data from multiple sites. At the same time, similar to complete pooling, the model has a fixed set of hyperparameters. Here, hyperparameters play the role of a joint prior over the parameters. They perform as a regularizer and prevent the model from overfitting on small batches.

Naive pooling is a variation of the complete pooling scenario (see Fig 2) in which the batch-effects in data are ignored by assuming that data in different batches are coming from the same distribution, *i.e*., $y_{1},\ldots ,y_{m}∼N\left(\mu ,\sigma^{2}\right)$ and we have:

$$\begin{array}{c} {\text{y}}_{i}=f_{\mu }\left(\text{X},\theta_{\mu }\right)+\epsilon \;\forall i\in \left\{1,\ldots ,m\right\}, \end{array}$$ (2)

where *ϵ* is zero-mean error with standard deviation $f_{\sigma }^{+}\left(\text{X},\theta_{\sigma }\right)$. Even though the naive pooling approach provides a simple solution to benefit from a larger sample size, the oversimplifying assumption on identical data distributions restricts its usage in normative modeling because batch-effects are reflected on the resulting statistics in Eq 1.

#### Pooling after data harmonization

In this approach, data are harmonized for batch-effects before pooling. Data harmonization overcomes the limitation of the naive pooling approach by adjusting the location and scale of the data for batch-effects. Hence, unlike naive pooling, assuming identical data distribution across batches is no longer a restrictive issue. Adopted from genomics, ComBat [30] is a popular method for harmonizing neuroimaging data. ComBat uses an empirical Bayes method for adjusting additive and multiplicative batch-effects in data. It has shown great potential in harmonizing different neuroimaging data modalities, including diffusion tensor imaging [31], cortical thickness [16, 32], and structural/functional MRI [33–35].

ComBat removes additive and multiplicative batch-effects while preserving the signal of interest in data:

$$\begin{array}{c} {\tilde{\text{y}}}_{i}=\frac{{\text{y}}_{i}-g\left(\text{X}\right)-\gamma_{i}}{\delta_{i}}+g\left(\text{X}\right), \end{array}$$ (3)

where ${\tilde{\text{y}}}_{i}$ is harmonized data that is expected to be homogeneous across batches; *γ*_*i*_ and *δ*_*i*_ are respectively the additive and multiplicative batch-effects. Here, *g*(**X**) is a linear or non-linear [35] function that preserves the signal of interest as specified in the design matrix **X**. After harmonization, Eq 2 can be used for modeling the pooled data.

However, ComBat (and in general data harmonization) has three potentially problematic theoretical shortcomings. We refer to these problems as theoretical because depending on the covariance structure of data they might or might not occur in practice, however, these problems are theoretically present due to restrictive implicit assumption of ComBat on the orthogonality of effects of interest and the noise in data (see A schematic illustration of ComBat in the S1 File). First, ComBat removes all variance associated with batch-effects and preserves *a priori* known sources of variation in data (which are accounted for in the design matrix **X**) and unknown sources of variation that are not correlated with batch-effects. In other words, it is necessary to specify in advance which shared variation should be retained. This requirement is restrictive especially when we are interested in an exploratory analysis of unknown biologically relevant factors (see Simulation study in the S1 File). An illustrative example is stratifying psychiatric disorders into subtypes [36]. Since subtypes are unknown in advance, their biological correlates in brain images can be removed or corrupted in the data harmonization process. Second, in many cases, clinical covariates (such as age) strongly correlate with batch-effects, thus, preserving the age effect may result in a partial presence of unwanted batch-effects in the harmonized data. Third, the harmonization process can make it difficult to interpret the data in the original scale of the data. For example, data are harmonised to have a single (average) variance across all sites, which is dependent on the sample characteristics (*e.g*., larger sites will have a greater contribution to the average variance), which can be potentially problematic if the sample characteristics change, or if the sites have different variances (*e.g*., due to heteroscedasticity across the range of the covariates). Moreover, harmonization needs to be done with care to avoid serious bias to downstream analyses [37].

Data harmonization via ComBat also suffers from a practical issue when adopted in the normative modeling life-cycle. ComBat requires access to data from all sites at the training time to compute the parameters *g*(**X**), and the variance of the noise. This drawback is problematic for updating the model parameters, model estimation on decentralized data, and model adaptation to local data. Because in these scenarios, we may not have access to all data due to data anonymity concerns or a lack of ethical permission for data sharing [17]. Recently, [35] presented an ad-hoc solution to this problem in a web application. This method is based on demeaning and rescaling the data from a new site using respectively the mean and standard deviation of residuals. However, the effectiveness of this approach in removing the batch-effects while preserving the signal of interest remained unexplored and needs further empirical evaluations.

#### Pooling with batch-effects as fixed-effect

In this setting, the batch-effects are directly used as covariates (in the design matrix *X*) in Eq 2. While effective in removing the batch-effects, this method suffers from the same theoretical and practical limitations of data harmonization. It regresses out the batch-effects, thus, part of the unknown but informative variance of interest in data that are correlated with batch-effects. Model adaptation and extension procedures are also restricted in this setting because it requires full data availability. In other words, since all sites need to be encoded in the design matrix at training time, it is difficult to deploy pre-trained models to new sites.

#### No-pooling

In the no-pooling scenario, we assume that the data in each batch are drawn from different distributions. Hence, a separate and independent set of model parameters are estimated for each batch (see Fig 2):

$$\begin{array}{c} {\text{y}}_{i}=f_{\mu_{i}}\left(\text{X},\theta_{\mu_{i}}\right)+\epsilon_{i}\;i\in \left\{1,\ldots ,m\right\}. \end{array}$$ (4)

No-pooling is immune to the theoretical problems of fixed-effect pooling and harmonization because the batch-effects are not directly removed from the data. However, it cannot take full advantage of the richness of big data. It is prone to overfitting, especially when $f_{\mu_{i}}$ and $f_{\sigma_{i}}^{+}$ are complex functions and the number of samples in each batch is small. This may result in spurious and inconsistent estimations of parameters of the model across different batches.

### A solution: Partial-pooling using hierarchical Bayesian regression

To overcome the aforementioned shortcomings, we propose a partial pooling approach based on hierarchical Bayesian regression (HBR) as a possible solution for completing the life-cycle of normative modeling.

HBR is a natural choice in modeling different levels of variation in data [23]. In HBR, the structural dependencies between parameters are incorporated in the modeling process by coupling them via a shared prior distribution over parameters. To adopt HBR for multi-site normative modeling, we assume $\theta_{\mu_{i}}$ and $\theta_{\sigma_{i}}$ in Eq 4 (that govern the data generating process for each batch **y**_*i*_) are coming exchangeably from the same prior distribution, *i.e*., $\forall i,\theta_{\mu_{i}}∼N\left(\mu_{\theta_{\mu }},\sigma_{\theta_{\mu }}^{2}\right)$ and $\theta_{\sigma_{i}}∼N\left(\mu_{\theta_{\sigma }},\sigma_{\theta_{\sigma }}^{2}\right)$ (see Fig 2). To this end, we use a wide Gaussian distribution as a weakly-informative hyperprior over parameters of the priors ($\mu_{\theta_{\mu }},\mu_{\theta_{\sigma }}∼N\left(0,10^{3}\right)$ and $log\left(\sigma_{\theta_{\mu }}\right),log\left(\sigma_{\theta_{\sigma }}\right)∼N\left(0,2.5\right)$). This choice provides a fair balance between the flexibility of the model and its computational speed because:

1. it is conjugate with likelihood and provides more computational efficiency in the sampling process compared to a non-informative uniform hyperprior;
2. given our limited prior knowledge about the distribution of parameters in the development phase, it improves the model’s flexibility compared to informed hyperprior (i.e., a narrow Gaussian distribution) when applied to different IDPs. Please note that the distribution of parameters (e.g., intercept and slopes in the linear case) can be very different from one phenotype to another, therefore, using an informative hyperprior could result in poor or biased parameter estimation;
3. such a joint Gaussian hyperprior acts like a regularizer over model parameters (similar to ridge regression) and prevents the model from overfitting on small batches. This feature is crucial in unbalanced data distribution settings when we have some sites with smaller sample sizes (see results in section Few-shot learning on small data).

Furthermore, HBR allows for a reasonable compromise between the complete pooling and no-pooling scenarios in the stability-flexibility spectrum as it combines all models in Eq 4 into a single model that benefits from the wealth of big data, thus results in more stable models. At the same time, like no-pooling, it estimates a separate set of parameters, thus different *f*_*μ*_ for and $f_{\sigma }^{+}$ for each batch (or group). Then in the normative modeling setting, the deviations (z-statistics) for the *i*th batch are computed as follows:

$$\begin{array}{c} {\text{z}}_{i}=\frac{{\text{y}}_{i}-f_{\mu_{i}}\left({\text{X}}_{i},\theta_{\mu_{i}}\right)}{f_{\sigma_{i}}^{+}\left({\text{X}}_{i}.\theta_{\sigma_{i}}\right)}. \end{array}$$ (5)

By using separate *f*_*μ*_ and $f_{\sigma }^{+}$ across batches, the z-statistics are respectively compensated for the additive and multiplicative batch-effects without the need to harmonize data primarily. Therefore, they accommodate batch-effects thorough modelling them explicitly in the generative model. In addition, unlike harmonization, HBR does not directly remove batch-related variability from data, thus, it preserves unknown sources of biological variations that correlate with batch-effects in data (see Simulation study in the S1 File).

HBR also presents several appealing features that make it the first choice for sustainable normative modeling. The generative nature of the model and shared prior distribution over parameters facilitate the model extension and adaptation, especially when dealing with decentralized data. Hence, it fulfills the technical requirements of normative modeling life-cycle. Furthermore, HBR provides the possibility to account for more than one group-effect and as a result more than one batch-effects in data. This is a favorable feature when we intend to simultaneously deal with several batch-effects in data (for example variability in both scanners and preprocessing software). In addition, it provides the possibility to include other informative group-effects (such as sex and ethnicity) in the hierarchical modeling process of the HBR.

#### Model extension using HBR

**Fig 3:**
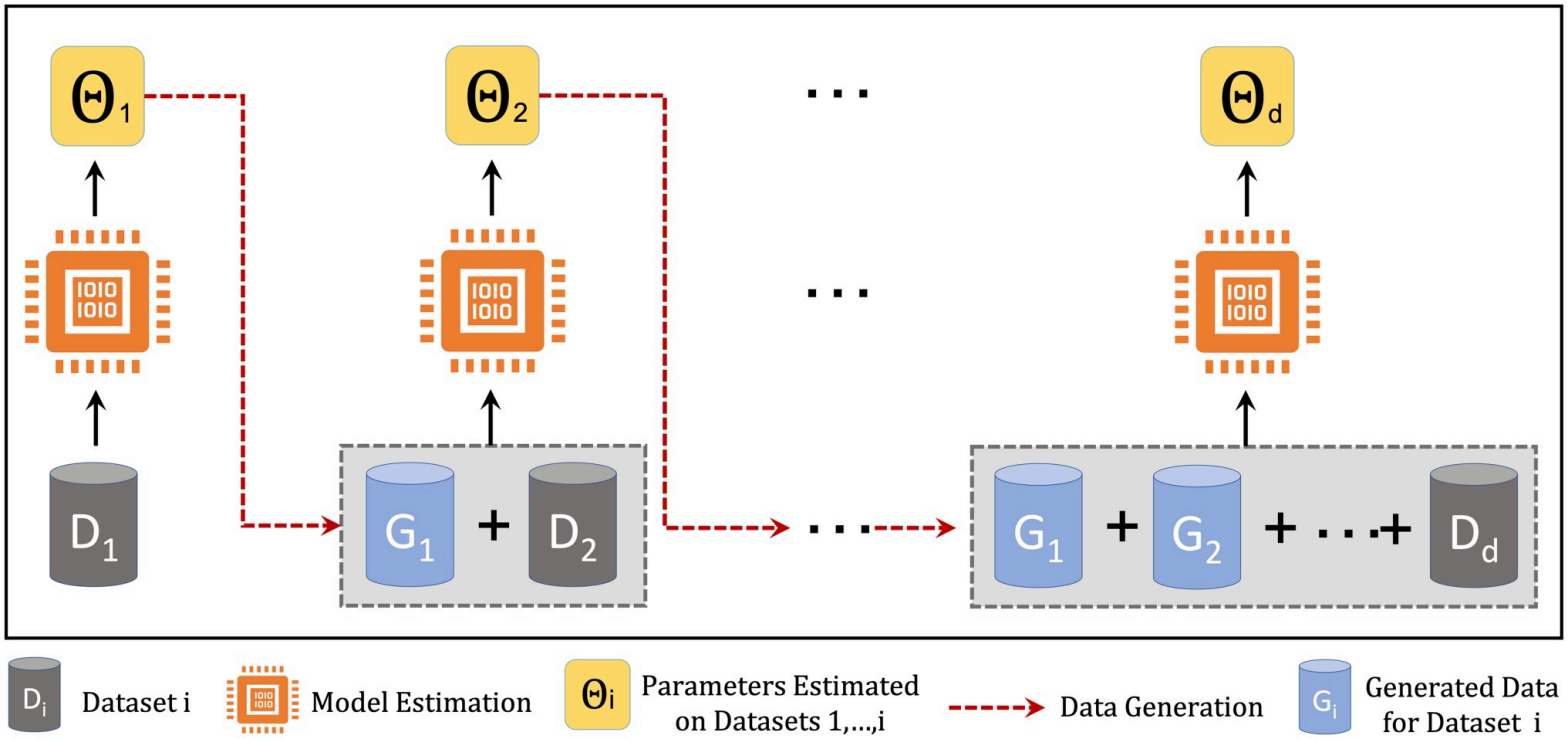
Model extension loop in multi-site normative modeling using HBR. The synthetic data generated for stages 1, …, *i* − 1 are used to estimate the model parameters at stage *i*. Model extension provides the possibility of updating the model parameters over time, and parameter estimation on decentralized data.

Considering the Bayesian nature of the HBR, once the parameters and hyperparameters of the model for a specific brain measure **y**_*i*_ are inferred, we can use the generative nature of the model to simulate synthetic neuroimaging measures ${\hat{\text{y}}}_{i}$ by sampling from the posterior predictive distribution of the model. In the normative modeling context, each generated sample represents the data for a single healthy participant. We exploit this property to implement the extension loop in the model development process. The model extension loop in Fig 1 can be expanded to a repetitive process of data generation and model estimation as illustrated in Fig 3. Here, we assume that we have access to the data from a single dataset at stage *i* of the model estimation. To estimate the model parameters at stage *i*, the synthetic data generated for 1, …, *i* − 1 stages are used to set up the complete dataset for parameter estimation.

In this scheme, if each stage is defined as a time interval, then the model extension loop can be used to update the model parameters over time and when new datasets are available. On the other hand, if each stage is defined as the geographical data distribution across data centers, then the model expansion loop can be used to train a reference normative model on decentralized data. These characteristics are crucial to maintaining the life-cycle of normative modeling.

#### Model adaptation by transferring parameters

Importantly, HBR also provides the possibility to transfer the knowledge inferred about the distribution of hyperparameters from a primary set of observed data **y** (in the model development process) to secondary datasets from new sites y* when deploying model at local centers. To achieve this, we propose to use posterior distributions of hyperparameters of the reference normative model, *i.e*., $p(\mu_{\theta_{\mu }}|\text{y})$, $p(\sigma_{\theta_{\mu }}|\text{y})$, $p(\mu_{\theta_{\sigma }}|\text{y})$, and $p(\sigma_{\theta_{\sigma }}|\text{y})$, as *informative* hyperpriors for the secondary model. Informative hyperpriors enable us to incorporate pre-existing evidence when re-inferring the model on new data rather than ignoring it when using non-informative or weakly informative hyperpriors. This strategy can be seen as an inductive transfer learning strategy [26] (by transferring knowledge of parameters) in which the source domains are the same (i.e., the covariates), but the target domains are different but related (neuroimaging data from multiple sites).

The proposed parameter transfer learning approach enables effective model adaptation to local data without having access to the primary data which is used to estimate the reference model. Considering we do not need to access the local data in the development phase, no data transfer between the development and deployment nodes is required, and only model parameters are exchanged. This feature is critical for privacy-preserving model portability in the federative learning setting.

We emphasize that the model adaptation is different from the model extension process. The model extension is used during the reference model development in which we aim to derive a larger model from a smaller one. Whilst model adaptation is used in the model deployment, where we aim to distill a smaller model from a reference model on local data.

### Anomaly detection in normative modeling

**Fig 4:**
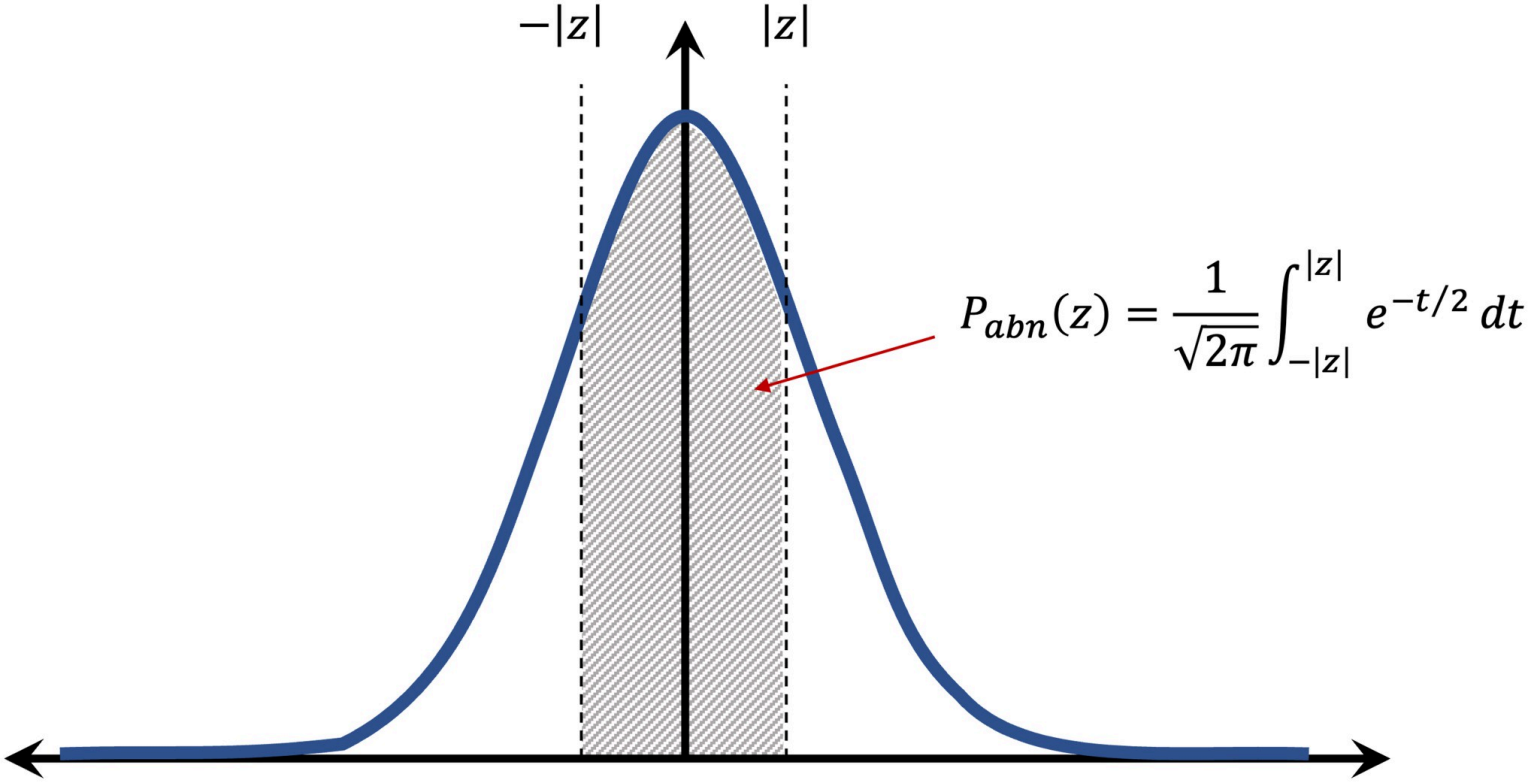
The area of the shaded region is computed as the abnormal probability index *P*_*abn*_ for a given z-score *z* (i.e., the deviation from norm of population). The *P*_*abn*_ is zero for a sample with 0 deviation from the norm and is getting closer to 1 as |*z*| grows.

The core aim of normative modeling is to derive the normative range for a structural or functional brain measure. Therefore, we only need data from healthy participants to derive the model (although normative models can also be estimated from other populations). This property is advantageous given the excess of data availability for healthy populations compared to clinical populations. If successful, then any large deviation from this normative range is interpreted as an abnormality in the brain that can be studied concerning different mental disorders. Given the normal distribution of z-scores and without any assumption on the direction of abnormal samples (left or right tail), these abnormalities can be quantified in the form of a probability by computing the area of the shaded region in Fig 4. To this end, each z-score *z* ∈ **z** in Eq 1 can be mapped to its corresponding abnormal probability index *P*_*abn*_(*z*) as follows (for more details on derivation see appendix Calculating the abnormal probability index in the S1 File):

$$\begin{array}{c} P_{abn}\left(z\right)=\frac{1}{\sqrt{2\pi }}\int_{-|z|}^{\left|z\right|}e^{-t^{2}/2}dt=\frac{2}{\sqrt{2\pi }}\int_{-\infty }^{\left|z\right|}e^{-t^{2}/2}dt-1, \end{array}$$ (6)

where, $\frac{1}{\sqrt{2\pi }}\int_{-\infty }^{\left|z\right|}e^{-t^{2}/2}dt$ is the cumulative distribution function of the normal distribution at |*z*| and can be easily computed. *P*_*abn*_ is zero for a sample with 0 deviation from the norm and is getting closer to 1 as |*z*| grows. This index can be employed to detect anomalies in brain measures in an anomaly detection scenario [38, 39]. This approach, in combination with normative modeling, provides an effective tool for data-driven biomarker discovery (see results in section The deviations are distinctive).

### Experimental materials and setups

In this section, we describe the experimental data and four experiments that are used for evaluating HBR in the normative modeling life-cycle.

#### Datasets and preprocessing

Table 1 lists the 16 neuroimaging datasets that are used in our experiments. For the ABCD dataset [15], we used data from the first imaging timepoint for subjects included in the *v*2.0.1 curated release. For the UK Biobank (UKBB) study [10], we used approximately 15000 subjects derived from the 2017 release. For the Human Connectome Project aging, development and early psychosis studies (HCPAG, HCPDV and HCPEP, respectively) we used data from the 1.0 data release. Further details surrounding the other datasets can be found in the relevant papers listed in Table 1. Ethical approval for the public data were provided by the relevant local research authorities for the studies contributing data. All subjects provide written informed consent for their data to be used for the purposes reported in this manuscript. For the public datasets, if there are minors (*e.g*., under 18 years), then this consent was also provided by the parent or guardian. For the clinical data in the TOP dataset, approval was obtained via the Regional Committee for Medical Health Research Ethics South East Norway Approval number 2009/2485 − *C*.

**Table 1:** Demographics of multi-site experimental data. (*) The HCPDV and HCPAG datasets are collected by the same data acquisition centers. We consider this in computing the total number of scanners in data.

Datasets	No. Scans	No. Patients	No. Scanners	Age Range	Sex M/F	FS Version
ABCD [15]	10732	-	29	9–11	52%/48%	6.0
CAMCAN [40]	647	-	1	18–88	49%/51%	6.0
CMI [41]	893	-	2	18–88	62%/38%	6.0
CNP [42]	264	49(SZ),49(BD),41(ADHD)	2	21–50	56%/44%	6.0
FCON [43]	1021	25(ADHD)	18	8–85	43%/57%	6.0
HCP [44]	1113	-	1	22–37	46%/54%	5.3
HCPAG [45]	677	-	5*	36–100	43%/57%	6.0
HCPDV [46]	653	-	5*	8–22	49%/51%	6.0
HCPEP [47]	180	123(EP)	4	17–36	62%/38%	6.0
IXI [48]	557	-	1	20–86	44%/56%	6.0
NKI [49]	482	-	1	6–85	36%/64%	6.0
OASIS3 [50]	2044	271(DM),51(MCI)	5	43–97	42%/58%	5.3
OPN [51]	612	-	6	8–58	45%/55%	6.0
PNC [52]	1514	-	1	8–23	48%/52%	6.0
TOP [53]	823	167(SZ),193(BD),31(MDD),107(others)	1	17–69	53%/47%	6.0
UKBB [10]	14914	-	2	44–80	48%/52%	6.0
**Total**	37126	1107	79	6–100	49%/51%	-

**Fig 5:**
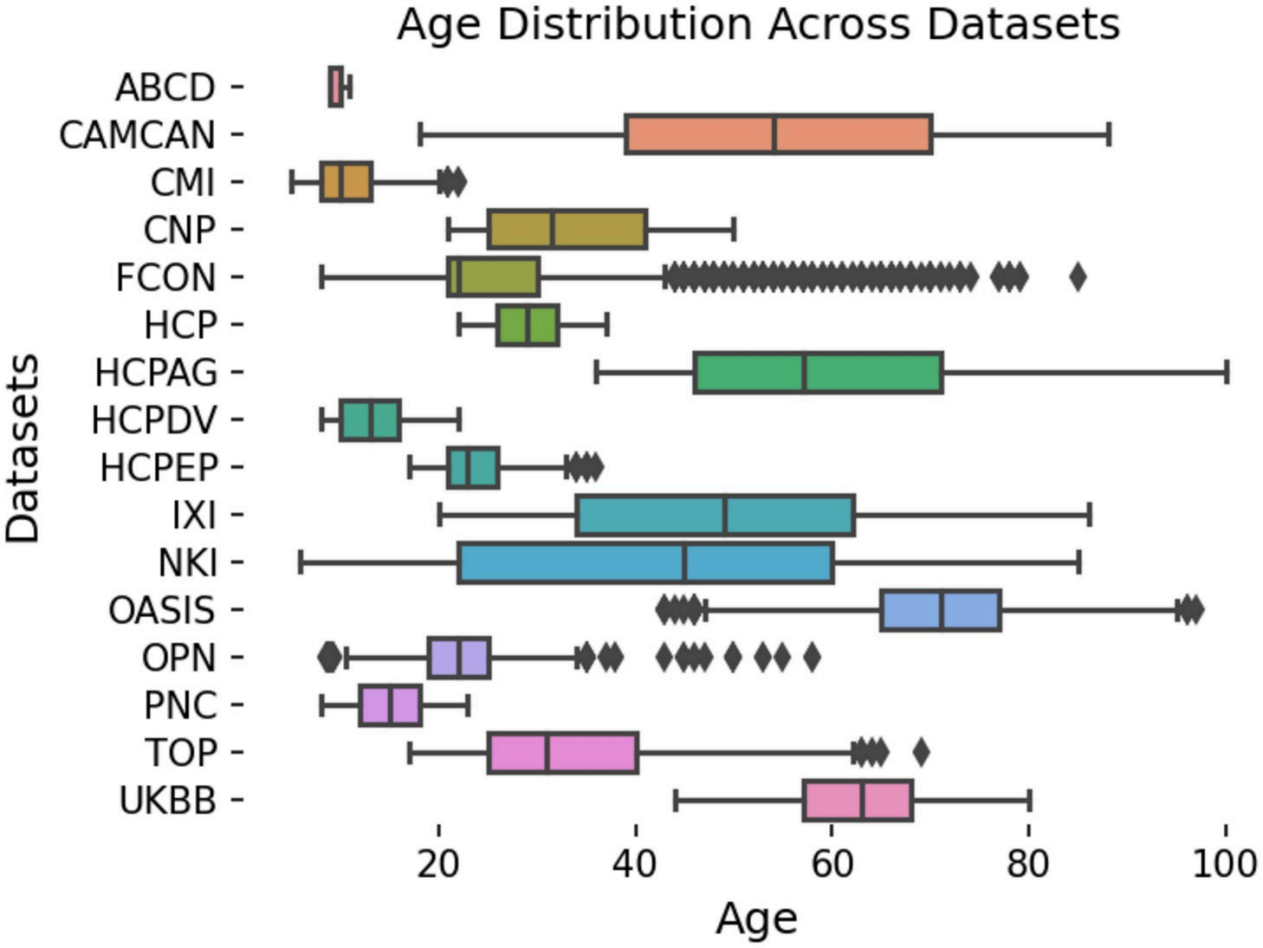
The age span of participants across 16 neuroimaging datasets. Our experimental data cover almost the full range of human life-span.

We have excluded participants with missing demographic (age/sex) information and those with poor quality imaging data. We excluded 1566 (4%) subjects due to low-quality images. Subjects were excluded if their scan-site median-centered absolute Euler number was higher than 25. The Euler numbers are computed as a part of standard recon-all Freesurfer [54] pipeline. The exclusion of outliers based on Euler numbers has been shown to be a reliable quality control strategy in large neuroimaging cohorts [55, 56]. Median centering is necessary because the Euler number is scaled differently for different datasets. The threshold of 25 was determined empirically by manually examining the excluded scans. The final data consists of 37126 scans from 79 scanners that reasonably cover a wide range of human lifespan from 6 to 100 years old. Fig 5 depicts the age span for each dataset. Note that the peak at approximately 10 years is driven by the ABCD dataset, where subjects are all nearly the same age. These properties make these data a perfect case-study for large-scale multi-site normative modeling of aging. The data also contain 1107 scans from participants diagnosed with a neurodevelopmental, psychiatric, or neurodegenerative disease, including attention deficit hyperactivity disorder (ADHD), schizophrenia (SZ), bipolar disorder (BD), major depressive disorder (MDD), early psychosis (EP), mild cognitive impairment (MCI), and (mild) dementia (DM).

**Fig 6:**
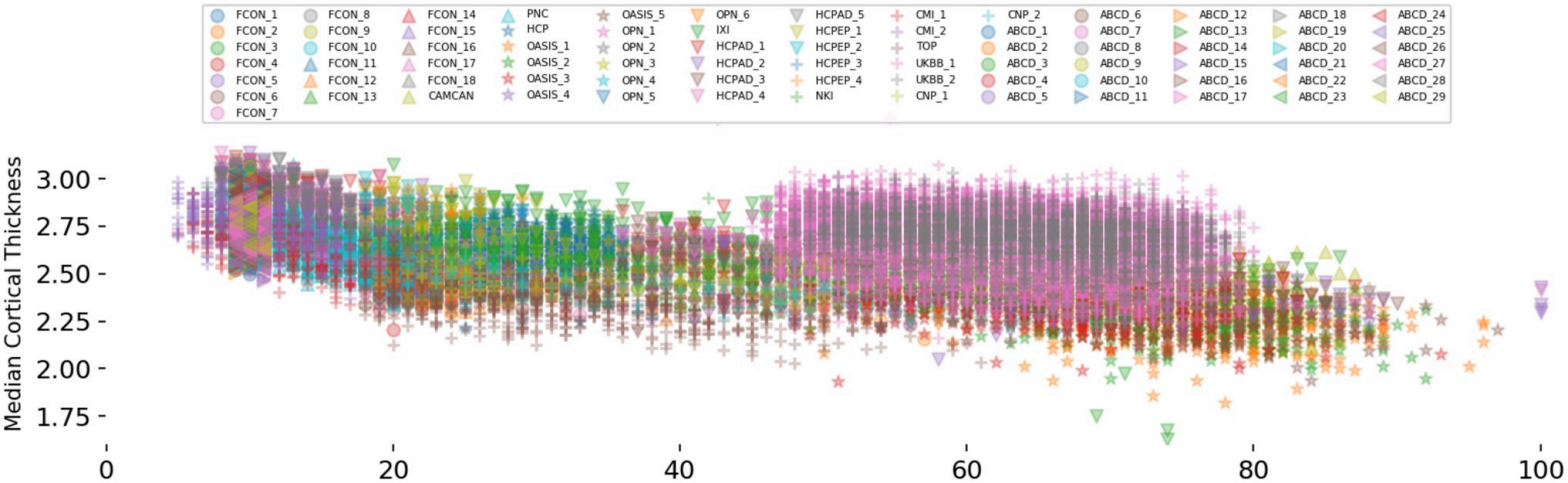
The distribution of median cortical thickness with respect to age across 79 scanners in 16 datasets. While an overall effect of aging on cortical thinning is present, however, it is highly contaminated with site-effect. The data in some datasets (*e.g*., UKBB) show relatively higher cortical thickness compared to the others.

In our analyses, we use cortical thickness measures estimated by Freesurfer version 5.3 or 6.0 over 148 cortical regions in the Destrieux atlas [57]. We have two motivations for this choice: i) the site-effect is very salient in the cortical thickness across data from different sites; ii) the fact that the effect of aging on thinning the gray matter is well-studied in the literature concerning different brain disorders. These features in data help us to better validate the method presented in this study. Fig 6 shows the distribution of median cortical thickness with respect to age across participants and scanners. It clearly shows the presence of an overall effect of aging on cortical thinning and the site-effect in data. For example, the cortical thickness is on average higher in the UKBB dataset than in other datasets.

#### Experiments

To demonstrate the effectiveness of HBR in completing the life-cycle of large-scale normative modeling, we set up four experimental settings for predicting the cortical thickness across 148 cortical regions: 1) multi-site data regression, 2) model extension, 3) model adaptation, and 4) anomaly detection. In all experimental configurations, we use only age as a covariate except for the fixed-effect site modeling in which, by definition, the one-hot encoding of scanner ids are also included in the covariates. We use sex as a group-effect in all estimated models. In the HBR case, the scanner is also included as a group-effect. All experiments and evaluations are repeated ten times with different random healthy participants in the training and test phases.

In the multi-site data regression setting, the goal is to compare the performance of HBR with naive pooling, fixed-effect pooling, pooling after data harmonization, and no-pooling models in deriving the normative range of cortical thicknesses across 148 brain areas. Here, we assume the data from all scanners are available when estimating the normative model, *i.e*., a centralized data architecture. In each experimental run, 80% of healthy samples are randomly selected to train the regression model. The remaining 20% are used for the evaluation. We modeled the effect of age on the response variable in three ways, 1) as a linear effect, 2) as a non-linear effect using a cubic polynomial, 3) as a non-linear effect using a cubic B-spline basis set expansion with 5 evenly spaced knots. Given the characteristics of experimental data, we use a site-specific homoscedastic form for the variance. We emphasize that the proposed framework is capable of modeling heteroscedasticity. Here, using a heteroscedastic model for the variance did not provide any advantage at the cost of higher model complexity (for a comparison see HBR with heteroscedastic noise model in the S1 File). Three metrics are used to evaluate the quality of fits, i) Pearson’s correlation coefficient (RHO) between observed and predicted brain measures; ii) standardized mean squared error (SMSE), and iii) mean standardized log-loss (MSLL). In the latter two cases, the lower values for the metrics represent the higher quality of the fitted function. While correlation and SMSE evaluate only the predicted mean, MSLL also accounts for the quality of estimated variance which plays an important role in deriving deviations from the norm (see Eq 1).

In the model extension experiment, the goal is to compare the performance of the HBR models trained on the centralized and decentralized data. While in the former, we use the same configuration in the multi-site regression experiment, in the latter case, we assume that we have only access to one dataset at each time-step and we estimate the model parameters sequentially, *i.e*., adding one dataset at a time until it covers all datasets. We generated 5 samples for each age value (in the range of 10 to 90 years old) and each gender in the data generation process for each dataset (80 × 2 × 5 = 800 samples). The same evaluation metrics are used to compare these two different settings.

In the model adaptation setting, we demonstrate an application of HBR in a more realistic clinical scenario when the aim is to adapt the parameters of a reference normative model to private clinical data at local hospitals. To do so, we first use a linear homoscedastic model to estimate the parameters of the reference normative model on datasets with only healthy participants (ABCD, CAMCAN, CMI, FCON, HCP, HCPAG, HCPDV, IXI, NKI OPN, PNC, and UKBB). Then, in each run 50% of random healthy participants in clinical datasets, including CNP, HCPEP, OASIS3, and TOP are used to recalibrate the parameters of the reference model. The rest of the healthy participants and patients are used as test samples. It is important to emphasize that other methods including harmonization and complete pooling do not apply to this setting because they do not support model adaptation to new datasets. We compare the HBR with no-pooling in which separate models are trained for each clinical dataset.

In the anomaly detection experiment, we aim to exemplify a possible application of the full cycle of normative modeling (*i.e*., developing a reference normative model on a large healthy population and model adaptation to clinical data) in data-driven biomarker discovery. Here, we use the resulting z-scores in the model adaptation experiment in the anomaly detection scenario described in section Anomaly detection in normative modeling. The abnormal probability indices for each individual across 148 cortical regions are computed. Then, the region-wise areas under the ROC curves (AUCs) are derived to evaluate the predictive power of deviations for each diagnostic label. We employed a conservative approach to testing for statistical significance, where we performed permutation tests with 1000 repetitions and used false discovery rate (FDR) correction [58] to correct for multiple comparisons across 148 regions. To ensure the stability of results, only significant areas that pass the FDR correction in 9 or more out of 10 full experimental runs are reported. We refer to this as ‘significant and stable’.

#### Implementations and model settings

The HBR model is implemented in Python using the PyMC3 package [59]. A No-U-Turn sampler (NUTS) [60] is used for inferring the posterior distributions of parameters and hyperparameters. Normal and log-normal distributions are respectively used as hyperpriors for the mean and standard deviation of parameters of *f*_*μ*_ (see Fig 2). The distribution of the standard deviation of the homoscedastic noise in logarithmic space is set to a normal distribution with 0 mean and standard deviation of 2.5. Non-centered parameterizations are used to simplify posterior geometries and increase the performance of the sampler [61]. All implementations are available online within the PCNToolkit (v.0.18) package [62] at https://github.com/amarquand/PCNtoolkit. The high-performance computing techniques are employed in our implementations to parallelize the computations across computational nodes on a computer cluster.

For harmonizing data using ComBat, we use a Python implementation available at https://github.com/Warvito/neurocombat_sklearn. This implementation has the possibility to learn the ComBat parameters on the training data and apply them to the test data that is and essential feature for out-of-sample evaluations in our experiments. Age and sex are used in the design matrix (**X** in Eq 3) when applying the ComBat for data harmonization to ensure that their variability is preserved in data (see the distribution of data after harmonization in the S1 File).

## Results

### HBR, suitable flexibility for big multi-site data

**Fig 7:**
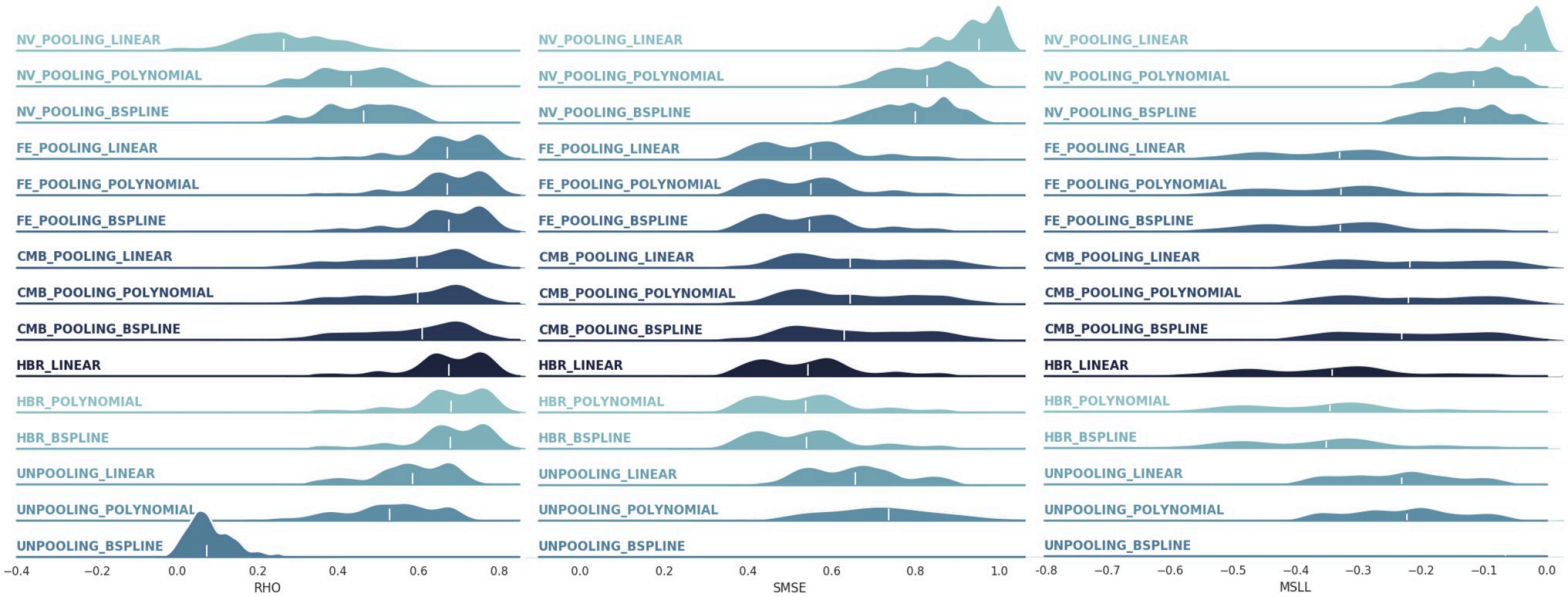
The distributions of correlation, SMSE, and MSLL across 148 cortical areas in the multi-site data regression. The white lines highlight the medians of distributions. Abbreviations: NV = naive, FE = fixed effects, CMB = ComBat, HBR = hierarchical Bayesian regression. The HBR and fixed-effect modeling show equivalently better regression performance compared to other approaches.

Fig 7 summarizes the empirical densities over three evaluative metrics (across 148 cortical areas) in the multi-site data regression scenario. Each column compares an evaluation metric across five modeling approaches (naive pooling (NV), fixed-effect pooling (FE), pooling after ComBat harmonization (CMB), HBR, and no-pooling); and three different model parametrization for the mean effect (linear, polynomial, and B-spline). In all cases, the HBR and fixed-effect modeling show equivalently better regression performance compared to other approaches. These two models both account for site-effect in data (unlike naive pooling), benefit from the richness of big data (unlike no-pooling), and have enough model flexibility to find the best fit to data (unlike naive pooling and pooling after harmonization). Even though they use different strategies to provide this flexibility; HBR by accounting for the difference between the distributions of signal and noise across multiple sites rather than ignoring or removing it, and the fixed-effect pooling by increasing the degree-of-freedom of the model via additional covariates (80 versus 1 for other models). However, this increased flexibility may result in an inferior performance when applied to small sample-size data. In addition, using batch-effects as covariates in the fixed-effect pooling method may result in regressing out informative but *a-priori* unknown variance from data.

On the other hand, on these experimental data, using more complex non-linear parameterizations has shown a negligible positive effect on the performances and linear models still provide competitive results. The very poor performance of the B-spline model in the no-pooling model (the SMSE and MSLL are out of range of plots) is the consequence of over parametrization on small sample size data. In short, our results show that choosing the right model flexibility on bigger data always results in more favorable regression performances. Therefore, taking an appropriate strategy for handling the site-effect in data can play a vital role in finding a better fit to data resulting in a better estimation of the normative range in normative modeling. Our experimental results confirm that HBR affords the proper model flexibility for modeling big multi-site data.

The distribution of measured metrics across 148 brain regions (thus 148 models) is multi-modal and wide in some cases, especially for MSLL measures. These diverse results across brain regions can be explained from data and model perspectives. From the data perspective, some brain regions might have a lower or higher relationship with covariates of interest (*e.g*., age). When there is no relationship between the two sides even the most complex models will fail if fairly evaluated. From the modeling perspective, in some brain regions, the model may not be able to explain the relationship between covariates and target brain measures. For example, when using linear models for modeling non-linear relationships. In this experiment, since all linear, polynomial, and B-spline models show similar performance, we conclude that the low performances (in MSLL, SMSE, and RHO) in some brain regions are due to the small effect of aging on the cortical thickness.

**Fig 8:**
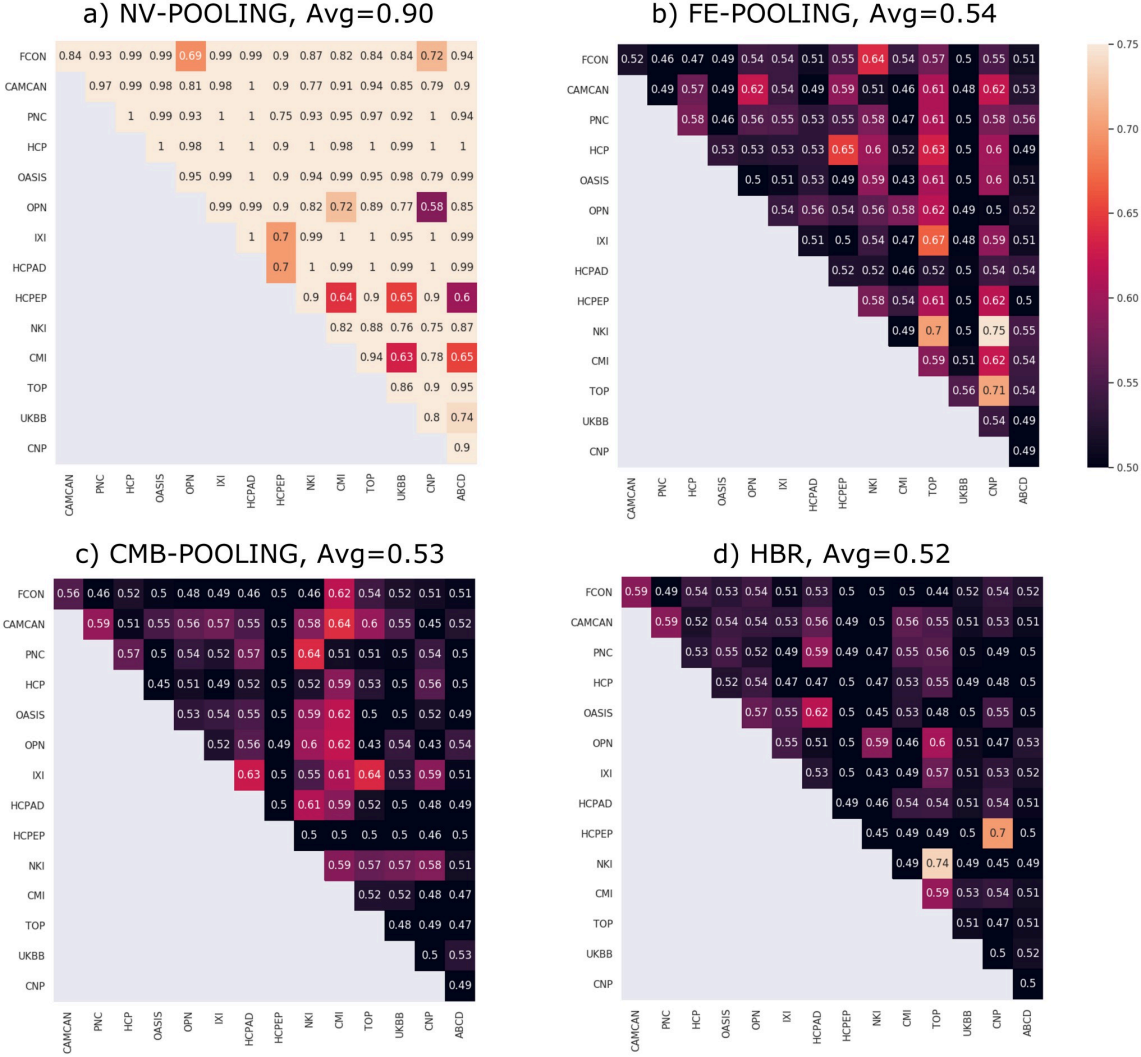
Balanced-accuracies in classifying z-statistics across different datasets in a one-vs-one scenario. The z-statistics are computed using different modeling approaches including naive pooling (NV-POOLING), fixed-effect pooling (FE-POOLING), pooling after ComBat harmonization (CMB-POOLING) and HBR. The results show that the site-effects are to high degree not present in the z-statistics in FE-POOLING, CMB-POOLING, and HBR.

We conducted an extra experiment to evaluate the performance of different techniques in removing site-effects in resulting z-statistics. In this experiment, the z-statistics are computed on the test sets across different modeling approaches. Then they are used as input to a linear support vector machine (SVM) classifier (C = 1) to classify the datasets in a one-vs-one scenario. The balanced-accuracies computed over 5-fold stratified cross-validation are reported in Fig 8. The meaningful difference between classifier performances of naive pooling (that ignores the site-effects) and the other methods (that use different strategies to exclude site-effects) demonstrates 1) the importance of correcting for site-effects; 2) the effectiveness of these different strategies to remove a majority of site variation in data (drop in the average accuracy from 0.90 to ∼0.53).

### HBR, distributed modeling on distributed data

**Fig 9:**
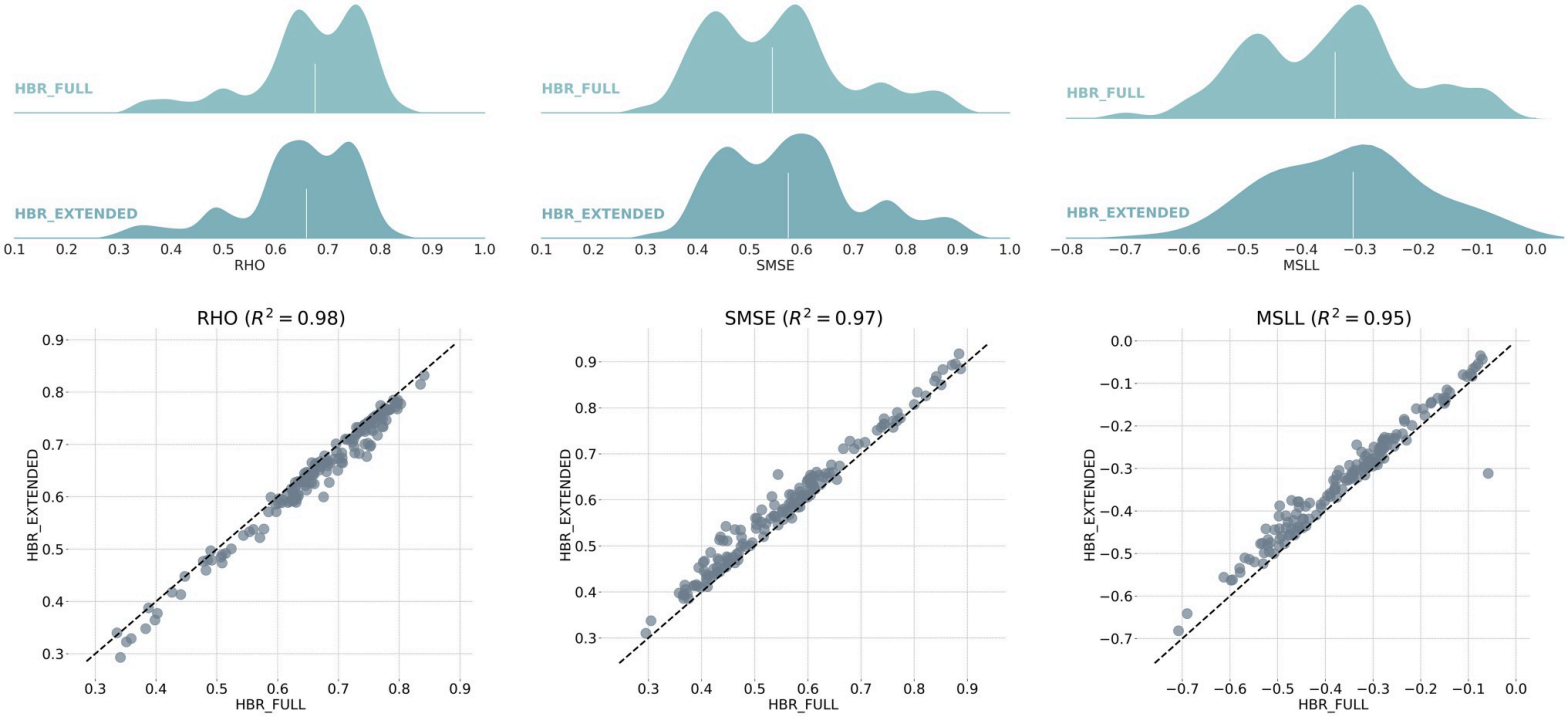
Comparison between the regression performance of HBR when trained on centralized data (HBR-FULL) versus decentralized model development using model extension strategy (HBR-EXTENDED). The ridge plots show distributions of correlation, SMSE, and MSLL across 148 cortical areas. As depicted in the scatter plots, HBR-EXTENDED models show very similar RHO (*R*^2^ = 0.98), SMSE (*R*^2^ = 0.97), and MSLL (*R*^2^ = 0.95) compared to HBR-FULL models trained on full data in one run.

Fig 9 compares the evaluation metrics for the linear HBR model with homoscedastic noise when trained on the centralized and decentralized multi-site data, adding one site at a time. The extended model shows very close RHO, SMSE, and MSLL compared to the model trained on full data in one run (*R*^2^ = 0.98, 0.97, 0.95, respectively). These results show the success of the proposed model extension strategy in estimating the mean prediction. However, the MSLL measure shows a slight but negligible decline in some regions; revealing the lower performance of the extended model in capturing the actual variance in some brain areas. Generating more samples in the data generation process might improve the model quality from this respect at the higher computational costs in time and memory. These promising results confirm the possibility of estimating multi-site normative models on distributed data across multiple data centers. This can significantly reduce the need for sensitive clinical data sharing. Furthermore, it reduces the data transfer, maintenance, and storage costs for storing several copies of the same data across several centers in centralized model development.

### Prior information matters

**Fig 10:**
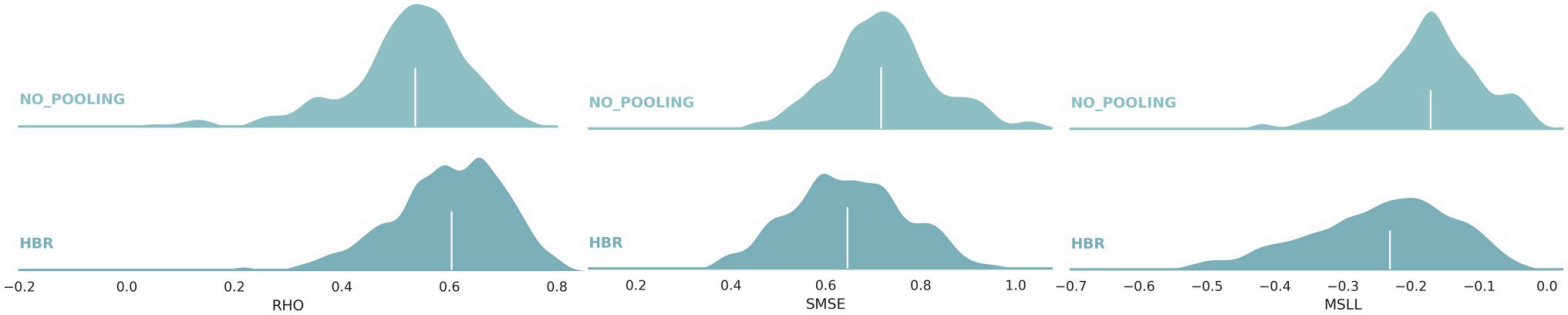
Comparing the regression performance of the adapted HBR model versus the no-pooling strategy. The ridge plots show distributions of correlation, SMSE, and MSLL across 148 cortical areas. The adapted HBR model shows a better regression performance compared to no-pooling.

Fig 10 compares the regression performance of the no-pooling approach with the adapted HBR model. While in the first case, we separately model the data from each presumably clinical center, in the second case, we try to benefit from transferring the knowledge from the reference normative model to local models. In all three evaluative metrics, the adapted HBR model shows a better regression performance compared to no-pooling. These results confirm the value of prior information learned by the reference model on big data in estimating more accurate normative models.

### The deviations are distinctive

**Fig 11:**
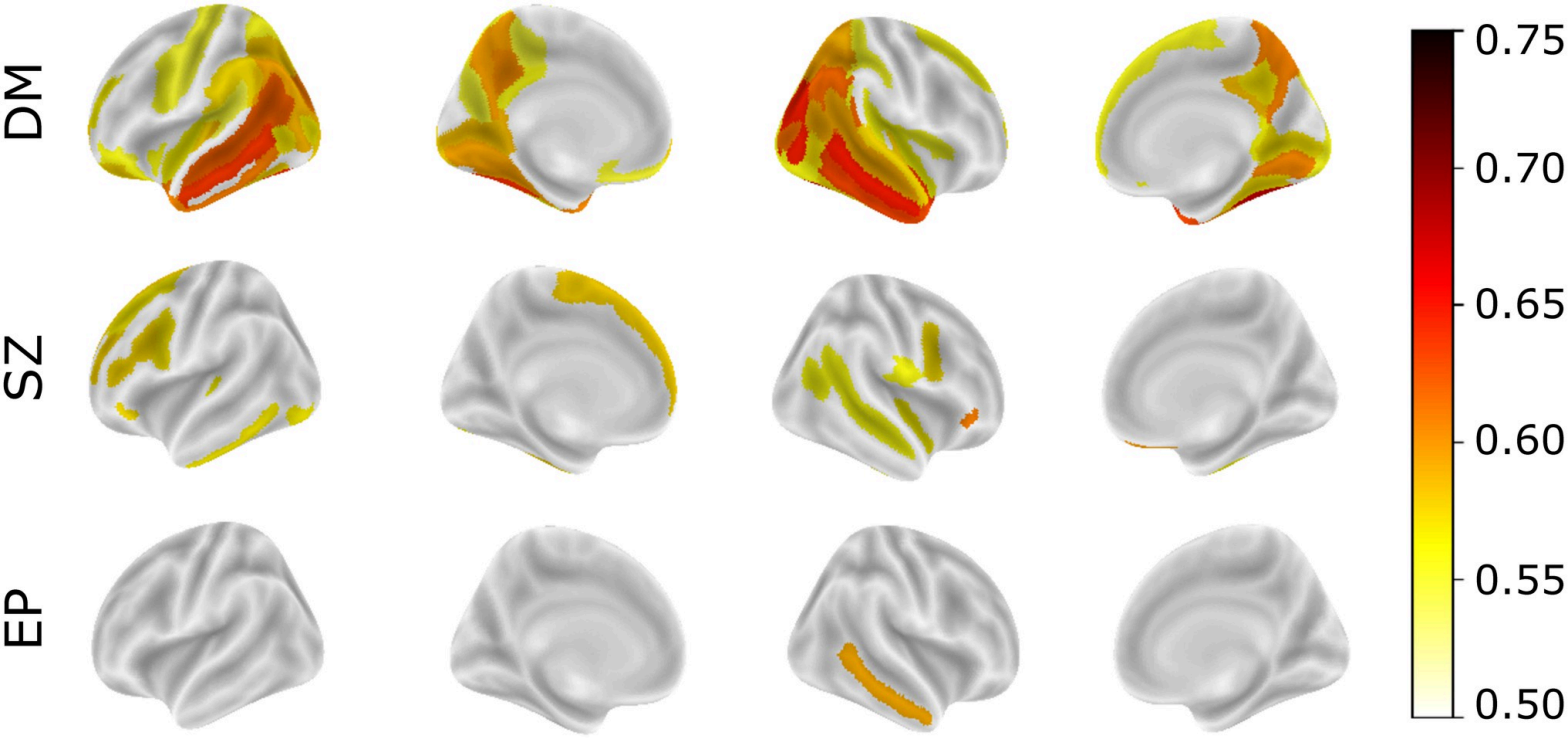
Significant and stable AUCs across brain regions for detecting healthy participants from patients in the anomaly detection scenario. In dementia (DM), the best performances are observed in the occipital and temporal lobes including bilateral occipitotemporal (fusiform) gyrus, right middle temporal gyrus, right superior/transverse occipital sulcus, right middle occipital gyrus, and left middle temporal gyrus. In schizophrenia (SZ), the distinctive areas are in the frontal lobe including the right orbital inferior frontal gyrus, right medial orbital sulcus, left superior frontal gyrus, left middle frontal sulcus, left anterior transverse collateral sulcus, and left inferior frontal sulcus. In early psychosis (EP), only the right middle temporal gyrus shows significant and stable AUC.

In Fig 11, we depict significant and stable AUCs across brain regions for different complex brain disorders and diseases. Here, the procedure explained in section Anomaly detection in normative modeling is used to derive the abnormal probability index for each sample and each region. Only significant and stable areas (see section Experiments for more detail on significance and stability criteria) are reported. Only the results for dementia, schizophrenia and early psychosis could pass our rigorous stability test.

**Fig 12:**
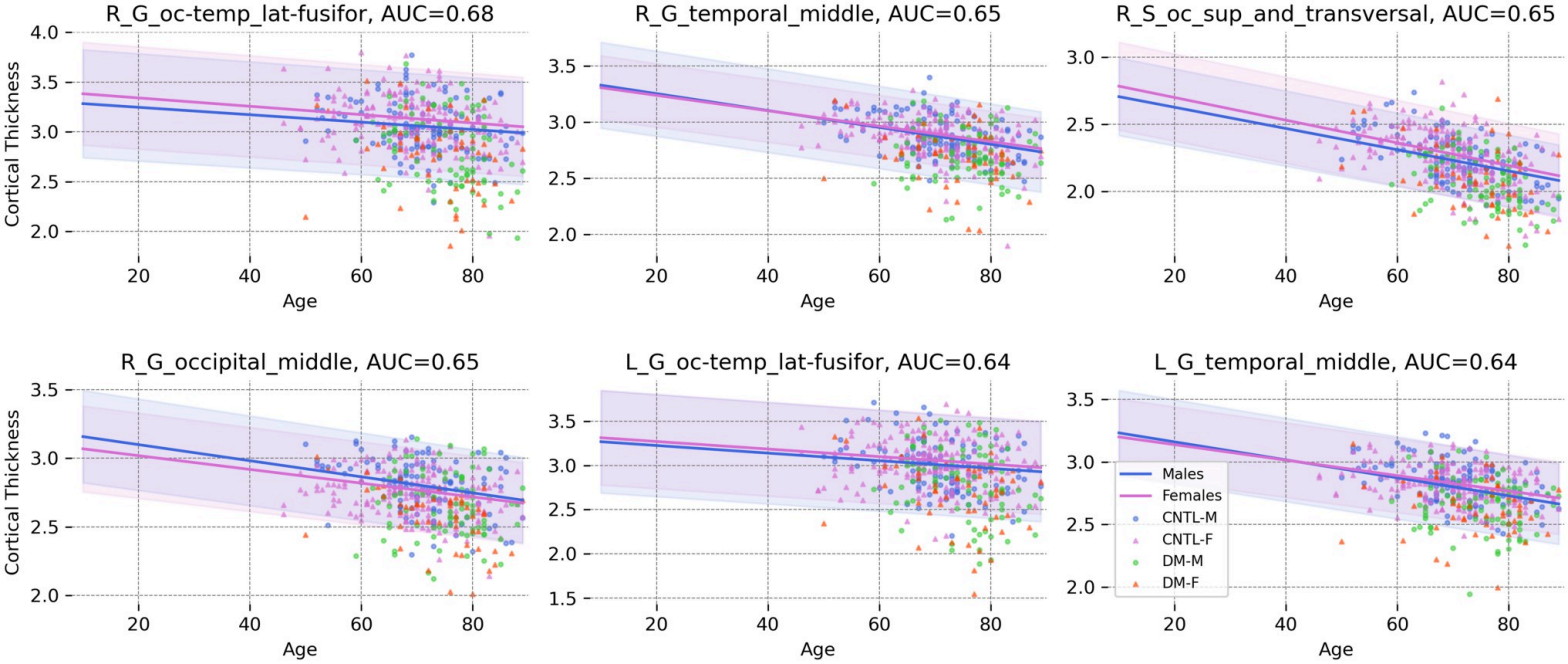
The norm and the 95% normative range for males and females in the 6 most distinctive cortical regions in dementia. Patients show lower cortical thickness than the norm of the population (Abbreviations: CNTL-F = healthy female, CNTL-M = healthy male, DM-F = female patient, DM-M = male patient).

In dementia cases, the best performances are observed in the occipital and temporal lobes including bilateral occipitotemporal (fusiform) gyrus (AUC = 0.68 and 0.64), right middle temporal gyrus (AUC = 0.65), right superior/transverse occipital sulcus (AUC = 0.65), right middle occipital gyrus (AUC = 0.65), and left middle temporal gyrus (AUC = 0.64). Fig 12 shows that patients with dementia manifest relatively stronger deviations in the respective brain regions. The proposed approach is capable of detecting brain regions that are repeatedly reported in the literature and have been linked to dementia [63–66]. Note that the patients with dementia were derived from the OASIS3 dataset, which contains only mild cases. Therefore, the accuracies reported are not directly comparable with studies derived from patients with more advanced forms of dementia.

**Fig 13:**
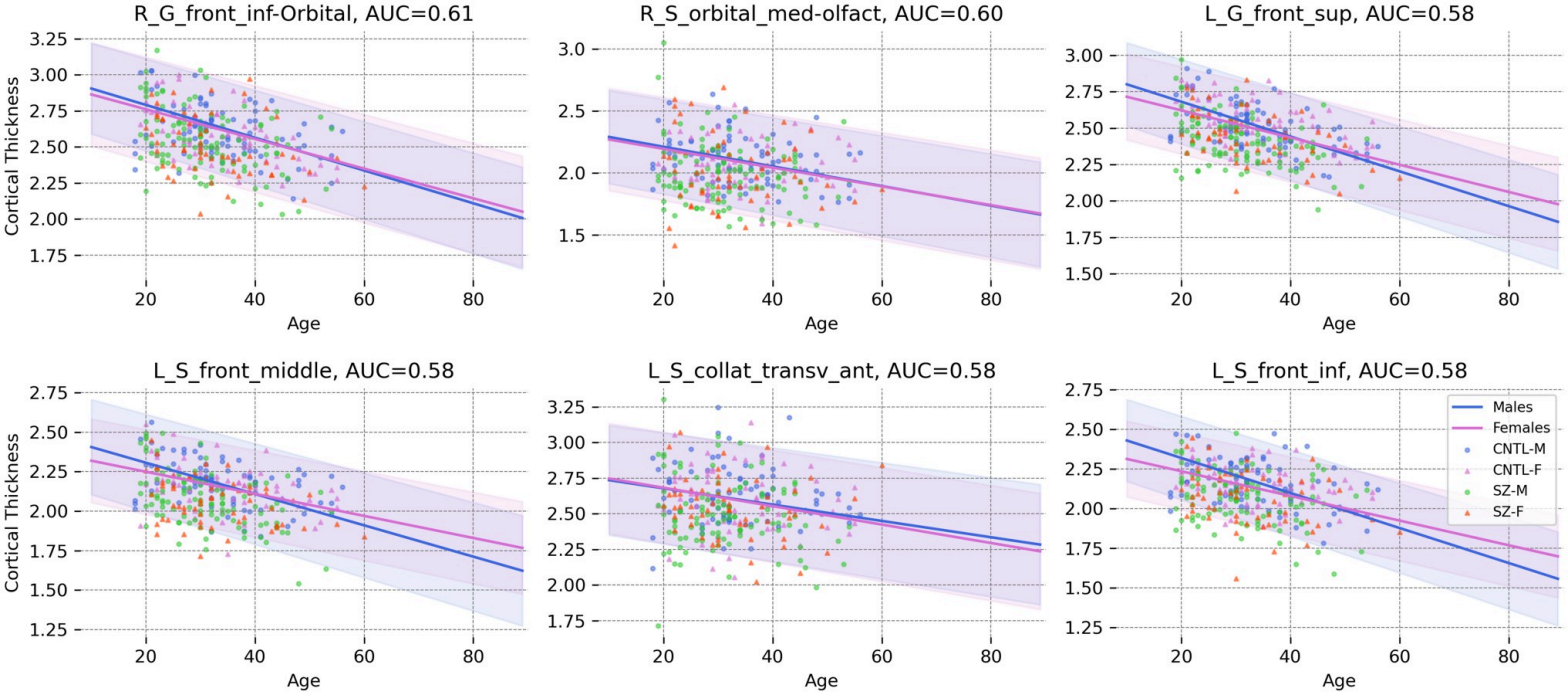
The norm and the 95% normative range for males and females in the 6 most distinctive cortical regions in schizophrenia. Patients show lower cortical thickness than the norm of the population (Abbreviations: CNTL-F = healthy female, CNTL-M = healthy male, SZ-F = female patient, SZ-M = male patient).

In patients with schizophrenia, our results show the concentration of distinctive areas in the frontal lobe including the right orbital inferior frontal gyrus (AUC = 0.61), right medial orbital sulcus (AUC = 0.60), left superior frontal gyrus (AUC = 0.58), left middle frontal sulcus (AUC = 0.58), left anterior transverse collateral sulcus (AUC = 0.58), and left inferior frontal sulcus (AUC = 0.58). These results are compatible with previous studies reporting cortical thinning in the frontal lobe in patients with schizophrenia [67–69]. Fig 13 shows how the cortical thicknesses of patients in these areas are distributed around the normative range. In early psychosis, only the right middle temporal gyrus (AUC = 0.59) shows significant and stable results. Cortical thinning in temporal regions has been reported in earlier studies on EP patients [70–72].

Even though these performances are lower than the state-of-the-art in classifying dementia and schizophrenia patients from healthy participants, it is important to consider the fact that our anomaly detection method is, in contrast, an unsupervised approach; in the sense that the model does not see any patient data during the training phase (*i.e*., in deriving the normative range).

Since patterns of sub- and supra-normal deviations can be extracted on an individual basis [4, 6, 73], our approach can be used as a tool for precision psychiatry by decoding the heterogeneity of complex brain disorders at the level of an individual patient [3, 74].

#### Few-shot learning on small data

**Fig 14:**
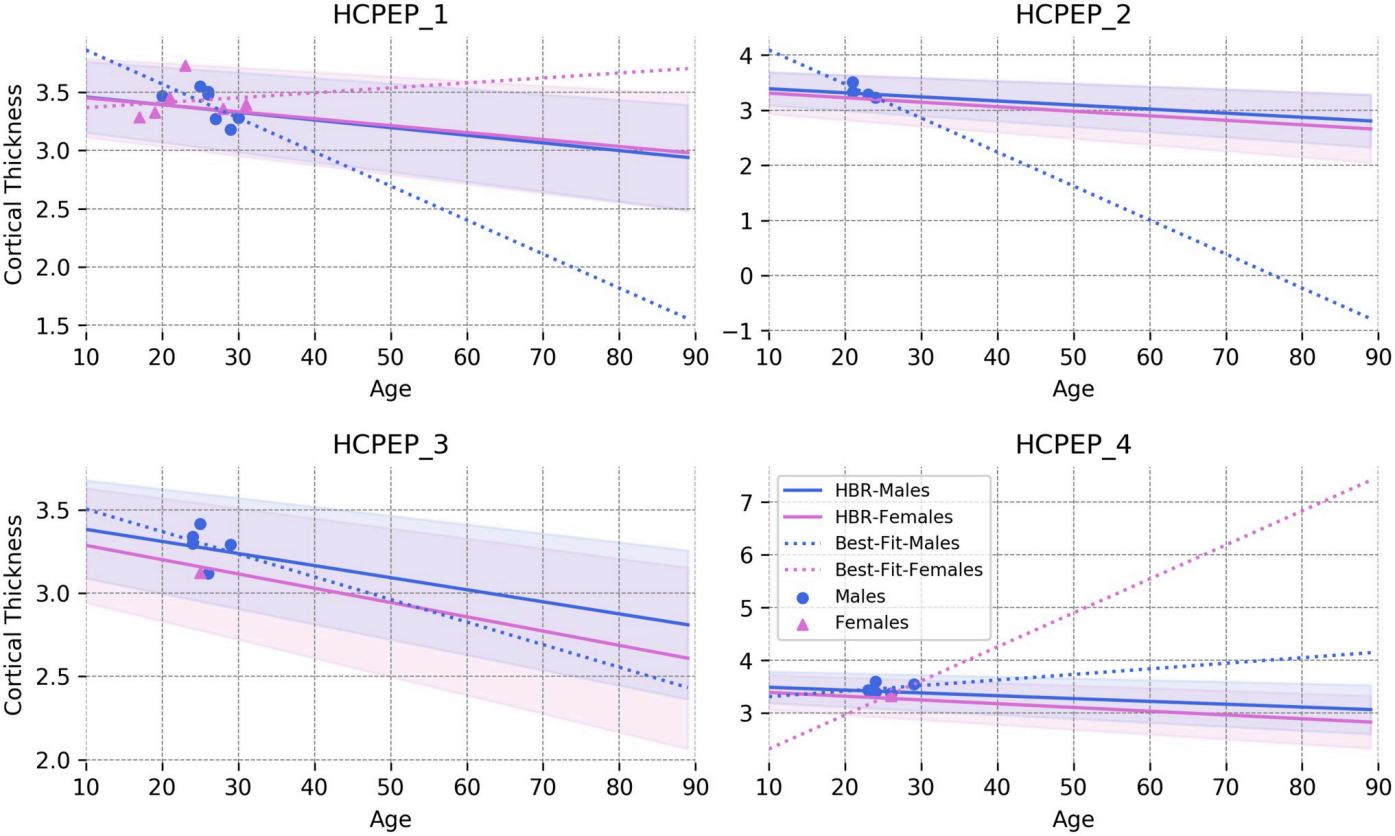
Normative ranges for the right middle temporal gyrus estimated by the adapted HBR model across four sites in the HCPEP dataset. The data points in the plots show the healthy subjects in the training set. The dashed lines show the best linear fit to the points for each sex. Benefiting from informative priors, the adapted HBR model provides a reasonable estimate of the normative range even on tiny training data.

Another appealing experimental observation in the model adaptation setting is the potential of the HBR model in learning reasonable normative ranges on tiny datasets. Fig 14 shows the normative range for the right middle temporal gyrus (the most distinctive region for early psychosis) across four different HCPEP acquisition sites. Only healthy subjects in the training set are depicted in the plots. In all sites, only a few training subjects are available for estimating the normative model. However, the HBR model can still find a reasonable estimation of normative ranges even for the extreme cases in the second and the third sites in which, respectively, 0 and 1 training samples are available.

The key feature of the HBR model that contributes to this performance is its informative prior that is inherited from the reference normative model. This informative prior is already learned from thousands of data points and acts as a high-level regularizer that prevents the model parameters from overfitting to small data. A possible example is the best linear fit for females (dashed purple line in the top left plot in Fig 14). Without having prior knowledge about the underlying effect of aging on the cortical thickness, the best linear estimate on the training data shows ascending trend for cortical thickness with aging. Another example is tiny training data with one or even zero samples. In these cases, estimating the parameters of the linear model is impossible; thus, prior knowledge about the problem plays a decisive role in finding reasonable estimations. We emphasize that this is not only a theoretical problem because, in practice, multi-site clinical datasets often have sites with few samples.

These results demonstrate the capabilities of the HBR model for few-shot learning [75] when adapting the reference model to very small local datasets. This feature can play even a more crucial role when adapting more complex normative models (for example, when *f*_*μ*_ and *f*_*σ*_ are parametrized on a neural network) on small data at local clinical centers.

## Discussion

Our positive experimental results demonstrate the success of hierarchical Bayesian modeling in fulfilling the technical demands for closing the life-cycle of normative modeling. In the following, we will discuss the methodological significance and the clinical relevance of our contributions. We further pinpoint the limitations of the proposed method and envisage possible directions for future enhancements.

### The methodological significance

Our HBR approach provides practical solutions to several key problems necessary to close the loop of normative modeling on realistic population-scale clinical datasets. Our main contributions are: i) accurately estimating centiles of variation whilst properly accounting for site variation with ii) manageable computational scaling to massive neuroimaging datasets; iii) a federated learning life-cycle that performs well on non-IID and unbalanced data and allows models to be updated as new datasets become available, without requiring access to the primary data and iv) enabling the transfer of information from population-level datasets to small clinical datasets.

We have designed our approach from the ground up with real-world clinical datasets in mind. The federated and distributed nature of our architecture is very important because it allows us to use large publicly available datasets for charting variation across the population to extract maximal value from clinical datasets that are often small and acquired on specific scanners. We consider model portability to be important for clinical applications. It is not feasible to transfer hundreds of thousands of scans to make predictions at a clinical site and –conversely– many clinical datasets are still small and can also be difficult to transfer (*e.g*., if subjects contributing data did not provide the necessary consent).

In this work, we have considered only models that are linear in the parameters. More specifically, for most of the experiments, we parameterized the HBR method as linear, allowing the estimation of a different slope for each scan site. While non-linear effects are seen in some neuroimaging derived measures [76]. Our results suggest that the linear model is sufficient for cortical thickness, and non-linear basis expansions did not explain more variance. However, for other neuroimaging-derived measures non-linear or heteroscedastic models may be more appropriate. We emphasize that our approach is fully modular, and such extensions can be easily integrated by adjusting the parametrization (see for example [77]).

### The clinical relevance

In our clinical application, we show that patients deviate significantly more from the estimated norm than healthy individuals (Figs 12 and 13) with a regional distribution of abnormalities that is largely consistent with the known pathology of each disorder. This is in line with earlier publications [4–7, 73], which show that while we observe differences between groups of patients and controls, those differences are not perfect to the extent of complete group separation [3]. This has been linked to the heterogeneous nature of these illnesses, which generally show a unique pattern of sub- and supra-normal deviations in individuals even when diagnosed with the same illness.

The ability of our approach to estimating normative models without the requirement to share sensitive data across different imaging and clinical centers can not be overemphasized in value as it allows us to map differences between individuals with a complex illness on a previously unprecedented scale. This is important as complex brain disorders are believed to have a unique manifestation across individuals [74]. Therefore, it is necessary to map those differences in large samples. Here, we provide a framework and tool that will allow us to extend this work in a principled fashion towards multi-center imaging studies such as the ENIGMA consortium [14]. While the present paper has a technical focus, we can already show that our method is capable to detect significant deviations from a normative process in individuals with a complex illness. Our contributions pave the way toward incorporating biological measures into the diagnosis and treatment of mental disorders to hopefully find the right treatment at the right time for the right patient.

### HBR versus data harmonization

In this study, we proposed an application of hierarchical Bayesian regression (HBR) for specific usage in federated multi-site normative modeling. In a normative modeling setting, we presented experimental evidence for the effectiveness of our method in deriving more accurate normative ranges and mitigating site-effects in resulting statistics. We showed how the HBR can be used as an alternative to data harmonization and fixed-effect modeling by resolving their theoretical and practical limitations in multi-site normative modeling on decentralized data. Nevertheless, we must emphasize that we do not consider HBR to be a data harmonization method, *per se*. Therefore, if the aim is merely data harmonization for other purposes than normative modeling then the HBR is not an appropriate choice because, whilst the z-statistics are cleared of site-effects, site-related variance is still present in the HBR predictions (*f*_*μ*_).

One of the important differences with respect to most harmonization techniques is that HBR enables estimating site-specific mean effects (*f*_*μ*_) and variations ($f_{\sigma }^{+}$) which are used in the normative modeling context to derive site-agnostic z-statistics. In contrast to most harmonization techniques, which often pool estimates over voxels or regions of interest, HBR pools over sites. This allows each site to have a different relationship with the covariates (*e.g*., different slopes or variances, as illustrated in S2 and S3 Figs in S1 File). This provides several advantages: first, it preserves differences across the range of the covariates (*e.g*., increasing variance with age across the lifespan in scenarios where age is correlated with site-effect), rather than forcing each site to have the same average variance. Second, it allows transfer learning to new sites, where the parameters are adjusted according to the characteristics of the new site and regularised by the informative prior distribution learned across the original sites, providing increased flexibility over (for example) harmonizing the data by applying the parameters learned on one set of data to a new dataset. This procedure is similar in spirit to meta-analysis as the second level parameters of the model (*θ*_*μ*_ and *θ*_*σ*_) and z-statistics are estimated for each site separately (but not independently). On the other hand, it is also similar to mega-analysis because the first level parameters (the parameters of the prior including $\mu_{\theta_{\mu }}$, $\sigma_{\theta_{\mu }}$, $\mu_{\theta_{\sigma }}$, and $\sigma_{\theta_{\sigma }}$) are estimated jointly across sites (See Fig 2).

In contrast, whilst harmonization provides the possibility to merge the data across different centers and perform the analysis on pooled data, this process might be harmful in the normative modeling context in which we are interested in the exploratory analysis of the variation in data. With this in mind, we do not claim HBR is a complete alternative to harmonization, and we recommend users choose the optimal approach according to their specific analytical goals.

### Limitations and future directions

The current implementation of HBR employs a Gaussian likelihood function, hence, assumes a Gaussian distribution for residuals. If this is not the case, the estimated centiles and z-scores might not be well-calibrated. Although this is usually not a big problem, distributions of some phenotypes are known to be skewed or bounded, and in these situations, this method would not provide accurate results. However, the presented HBR method is fully capable of accommodating non-Gaussian variability in data and we are exploring this in follow up work [78]. This possibility can be easily implemented by changing the likelihood and parameterize it over location, scale, and shape parameters (instead of mean and variance) [79]. Because we use a sampling approach for the inference, our method can estimate complex non-trivial posterior distribution with no closed-form analytical solutions. We are currently working on finalizing this extension. Another possible direction to solve this problem is to use likelihood warping [80], in which the data with arbitrary distribution is first warped into a Gaussian distribution and ordinary methods (with normality assumption) can be applied to derive the centiles of variation. Then these centiles are transferred back to the original distribution using a reverse operation.

Another limitation is that models are estimated separately for each brain region without accounting for correlations between brain regions. While this removes nearly all univariate site variation, our results in Fig 8 show that in all methods (including fixed-effect pooling, pooling after ComBat harmonization, and HBR), still, a few considerably above the chance-level performances are present in the tables. This is mainly because, in all benchmarked models, the harmonization and modeling are performed separately for each brain region without accounting for correlations between brain regions. Hence, only univariate site-effects are removed, and still, some multivariate site-effect might be present in data. Therefore, machine learning classifiers could still learn this residual information from the data [81]. One possible remedy for this problem is to harmonize the covariance structure of multivariate data [82]. Another option is to remove the batch-effects from machine learning predictions [81]. Of course, the presented anomaly detection method is immune to this deficit because it is separately applied to each cortical region. A conceptually straightforward extension to our model is to model correlations between brain regions that are related to site-effects in data. This extension can be straightforwardly integrated into the present model, for instance, using Wishart priors for the covariance between brain regions.

The quality of scans is a crucial factor in the success of developing a reference normative model. Low-quality noisy scans can impair the inference and result in inaccurate estimation of variability in data. Therefore, quality control (QC) is of high importance, especially in the normative modeling setting. While manual QC on massive datasets is costly and not practical, there is still no bullet-proof automatic QC method available in the field. For this study, based on recent studies in this area, we used FreeSurfer’s Euler number (that summarizes the topological complexity of the reconstructed cortical surface) as a criterion for the quality of scans, which shows a good correspondence with manual ratings of scan quality [55, 56]. Even though our manual inspection shows its reasonable performance, but we see addressing the open problem of developing automated QC as a decisive step toward reliable normative modeling, and we recommend this be given careful attention in future applications.

## Conclusions

In this study, we delineated the components involved in the life-cycle of normative modeling. We further elucidated the essential requirements of the normative modeling life-cycle to overcome the challenges imposed in the model development and deployment in real-world clinical applications. Then, we introduced a simple yet effective probabilistic federated learning approach to satisfy those requirements. The proposed hierarchical Bayesian regression method is quite flexible and accommodates a full range of parametric/non-parametric and linear/non-linear functions for modeling the signal mean and homoscedastic/heteroscedastic variance. On massive experimental data and in realistic scenarios, the HBR showed superior performance in deriving normative ranges of cortical thicknesses compared to its alternatives. In the longer run, we believe our methodological contributions provide a significant step toward bringing precision medicine to the diagnosis and treatments of complex brain disorders.

## Supporting information

S1 File (PDF)
