# Supplementary material for: Closing the life-cycle of normative modeling using federated hierarchical Bayesian regression
Source: PLoS One. 2022 Dec 8;17(12):e0278776. doi: 10.1371/journal.pone.0278776 (PMC9731431; doi:10.1371/journal.pone.0278776)
Supplement: S1 File — (PDF) [file pone.0278776.s001.pdf]

Supporting information

A schematic illustration of ComBat

Fig S1 schematically illustrates these two theoretical problems when adjusting neuroimaging data for batch-effects using ComBat. The signal variance in neuroimaging recordings consists of three main components: i) the known variance in data that we *a priori* are aware of their sources, thus, we can account for them when harmonizing data (using $g(\mathbf{X})$). Examples of these sources are age and sex of participants; ii) the unknown variance in data that might have some clinical relevance, however, we have no prior information about them. An example is subtypes in psychiatric disorders. And iii) the last component is the nuisance variance related to batch-effects in data, such as site-effects that we intend to remove from data. We emphasize that these three sources of variation often correlate with each other. For example, it is very common that the participants from a certain dataset have a specific age range. In this case, the site-effect correlates with age. These shared variances are depicted as overlaps between the circles in Fig S1.

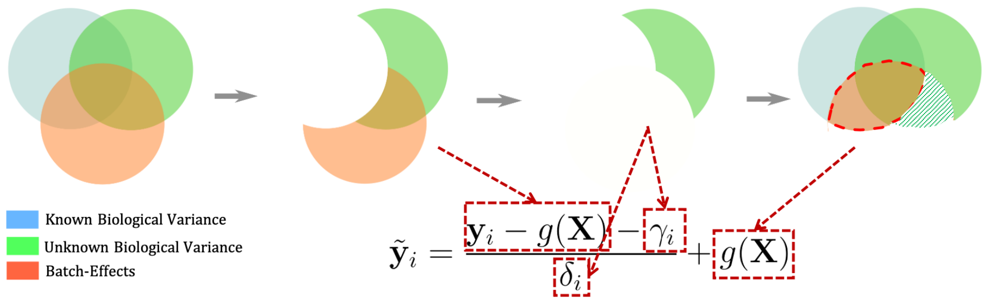

Fig S 1. A schematic illustration of steps in data adjustment process (Eq 3) in ComBat data harmonization. First, the biologically relevant variance, which is defined in the design matrix $\mathbf{X}$, is removed from standardized data in $\mathbf{y}_i$. Then, the additive (γ_i) and multiplicative (δ_i) batch-effects (here, to simplify the visualization we assume $\delta_i = 1, \forall i \in [1, \ldots, m]$) are removed; and in the end, the biologically-relevant variance are returned to the residuals. This process results in removing the shared variance between the batch-effect and unknown biological sources (the green striped area) while part of the batch-effect that correlates with known sources of variance is preserved in the harmonized data (the intersection between blue and red circles).

ComBat removes all variance associated with batch-effects and preserves *a priori* known sources of variation in data (which are accounted for in the design matrix $\mathbf{X}$) and unknown sources of variation that are not correlated with batch-effects. Since subtypes are unknown in advance, their biological correlates in brain images can be removed or corrupted in the data harmonization process (the green striped area in Fig S1). Moreover, in many cases, clinical covariates (such as age) strongly correlate with batch-effects, thus, preserving the age effect may result in a partial presence of unwanted batch-effects in the harmonized data (the intersection between red and blue circles in Fig S1). Moreover, in many cases, clinical covariates (such as age) strongly correlate with batch-effects, thus, preserving the age effect may result in a partial presence of unwanted batch-effects in the harmonized data (the intersection between red and blue circles in Fig S1).

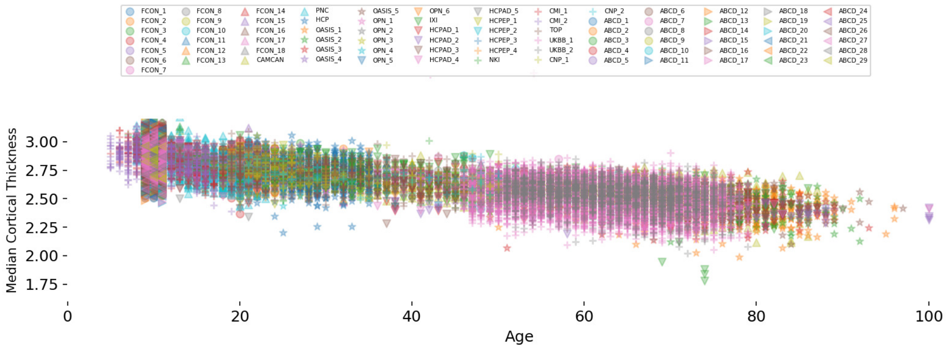

Fig S 2. Median cortical thicknesses across 79 scanners after ComBat harmonization. The additive and multiplicative site-effects are successfully eliminated after harmonization.

Calculating the abnormal probability index

We can compute the abnormal probability index P_{abn} for a given z-score z by calculating the area in the shaded region in Fig 4:

$$P_{abn}(z) = \frac{1}{\sqrt{2\pi}} \int_{-|z|}^{|z|} e^{-t^2/2} dt.$$

However, the empirical calculation of this area is challenging. To resolve this issue, we can use the properties of the normal distribution to reformulate its calculation as follows:

$$\begin{aligned} P_{abn}(z) &= \frac{1}{\sqrt{2\pi}} \int_{-|z|}^{|z|} e^{-t^2/2} dt = 1 - \frac{1}{\sqrt{2\pi}} \int_{-\infty}^{-|z|} e^{-t^2/2} dt - \frac{1}{\sqrt{2\pi}} \int_{|z|}^{+\infty} e^{-t^2/2} dt \\ &= 1 - 2 \times \frac{1}{\sqrt{2\pi}} \int_{|z|}^{+\infty} e^{-t^2/2} dt = 1 - 2 \times (1 - \frac{1}{\sqrt{2\pi}} \int_{-\infty}^{|z|} e^{-t^2/2} dt) \\ &= \frac{2}{\sqrt{2\pi}} \int_{-\infty}^{|z|} e^{-t^2/2} dt - 1 \end{aligned}$$

where, $\frac{1}{\sqrt{2\pi}} \int_{-\infty}^{|z|} e^{-t^2/2} dt$ is the cumulative distribution function of the normal distribution at $|z|$ and can be easily computed.

Data after ComBat harmonization

In pooling-after-harmonization models, we used ComBat for harmonizing data. The Python implementation at `https://github.com/Warvito/neurocombat_sklearn` is employed. Age and gender are used in the design matrix to ensure that their variability is preserved in data. Fig S2 shows the distribution of the median of cortical thicknesses across 148 brain regions. Compared to Fig 6 in the main text, it seems the scanner effect is successfully eliminated.

Simulation study

In this simulation study, we aim to experimentally illustrate the theoretical limitations of data harmonization in a normative modeling setting. Following the reasoning in section Pooling after data harmonization and Fig S1, we assume the signal $\mathbf{y} \in \mathbb{R}^{n \times 1}$ contains three components 1) the known source of variance that is measured via a covariate $\mathbf{x} \in \mathbb{R}^{n \times 1}$; 2) the unknown variance $\mathbf{z} \in \mathbb{R}^{n \times 1}$; and 3) the site-effect. In two

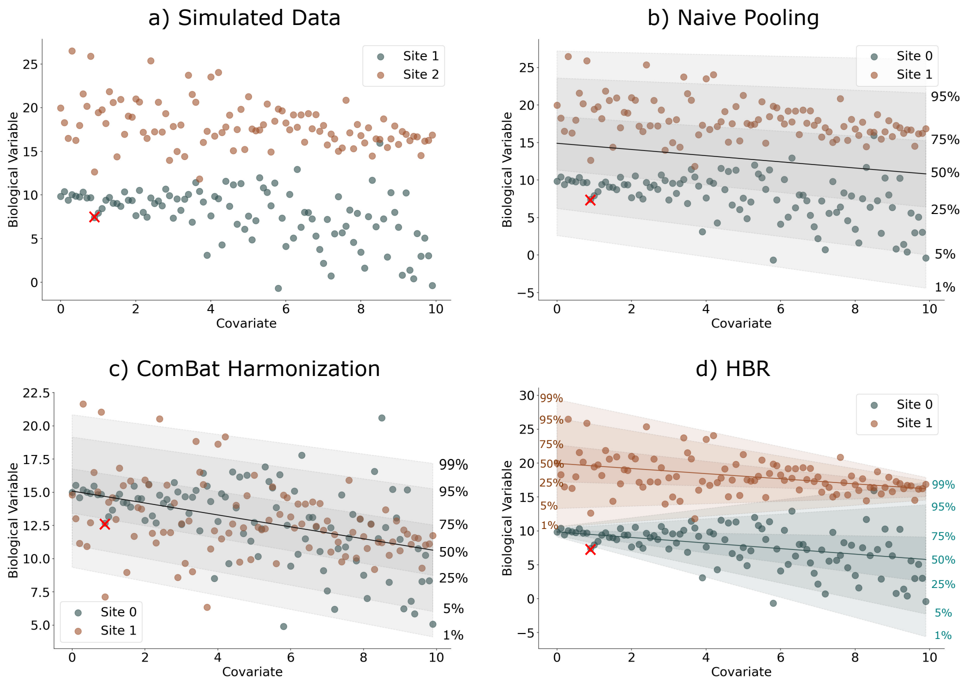

Fig S 3. a) The simulated data in the first simulation scenario in which a site-specific hidden factor affects the variability of the signal. The normative ranges are estimated using b) naive pooling, c) pooling after ComBat harmonization, and d) HBR. The estimated quantiles are misrepresented in the naive pooling case due to the site-effect. Even though the site-effect is removed after harmonization, the estimated quantiles are still misrepresented, and an outlier sample (marked by a red cross) remains within the normal range. This problem is solved when using HBR for normative modeling.

exaggerated simulation scenarios, we assume that a hidden site-specific factor is present in the data. We use the following function to simulate samples from two sites:

$$\mathbf{y} = a\mathbf{x} + b\mathbf{z} + c.$$

In the first scenario, we assume the hidden factor affects the variability of signal. Thus we set $a = -0.4$ and $\mathbf{x} \in [0, 10]$ as fixed for both sites (the effect of the measured covariate is fixed). We use c to simulated the additive site-effect and set it to 10 and 20 for *Site 1* and *Site 2*, respectively. On the other hand, we respectively set $\mathbf{z}$ to $(\mathbf{x}+2)/3$ and $(12-\mathbf{x})/3$ for *Site 1* and *Site 2*, and assume $b \sim \mathcal{N}(0, 1)$. This results in different heteroscedastic variations across two sites. The resulting simulated data are depicted in Fig S3(a). We used naive pooling, pooling after data harmonization using ComBat, and HBR methods to model the signal with respect to the covariate. In all cases, a linear model with heteroscedastic noise is employed. The results are shown in Fig S3(b-d). As expected, the quantiles estimated using the naive pooling approach are highly contaminated with the site-effect and are completely misrepresented. Therefore, the resulting z-scores are highly confounded with the site-effect. We further used a linear SVM classifier in 5-fold cross-validation to classify the z-scores into sites that results in 0.98 ± 0.02 balanced accuracy in the naive pooling scenario. This is while the site-effect is not present in the resulting z-scores after harmonization and using the HBR method (0.42 ± 0.07 and 0.48 ± 0.07 respectively). However, the estimated quantiles in the case of harmonized data are misleading. For example, an outlier sample (marked by a red cross) lies in the 25% quantile. This is while in the HBR case, the quantiles are estimated on

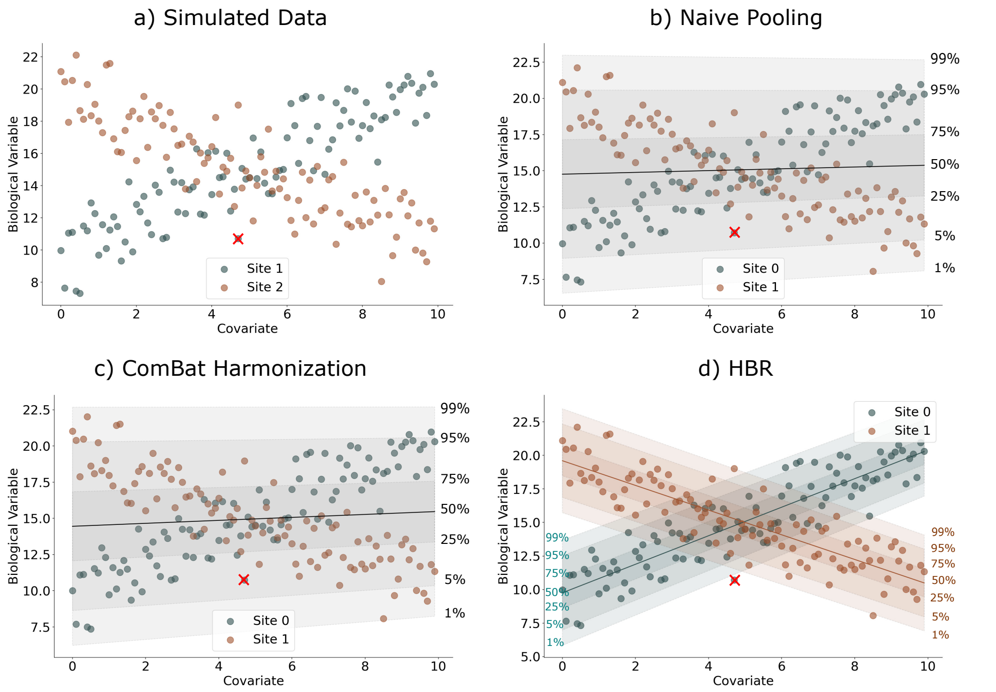

Fig S 4. a) The simulated data in the second simulation scenario in which a site-specific hidden factor affects the slope of the signal. The normative ranges are estimated using b) naive pooling, c) pooling after ComBat harmonization, and d) HBR. The estimated quantiles are misrepresented in both pooling scenarios because the ComBat fails in removing the site-effect. Thus, an outlier sample (marked by a red cross) is read within the normal range. This problem is solved when using the HBR for normative modeling.

original data and separately for two sites. Thus, the outlier sample remains outside the 1% centile.

In the second scenario, we assume the hidden factor affects the slope of the effect. In this case, we set $\mathbf{z}$ to 1.5 for both sites. Instead we use different slopes for two sites setting a to 1 and -1 for *Site 1* and *Site 2*, respectively. The rest of the parameters are set as they were in the first simulation. Here, the hidden factor correlates with both covariate and site-effect. The resulting simulated data are shown in Fig S4(a). The results of normative modeling using three different methods are shown in Fig S4(b-d). In this case, since the hidden factor correlates with the known covariate, the ComBat is completely disarmed, thus, the harmonization has almost no effect on the data. Thus, the quantiles estimated using both pooling approaches (with and without harmonization) are completely misrepresented. This is while the HBR provides a reasonably good estimate of quantiles for two sites.

Our simulations demonstrate how using data harmonization before normative modeling results in misleading quantile estimation, especially when a hidden variable correlates with site-effect and the covariate of the interest. Our analysis also highlights the differences between HBR and harmonization with ComBat. Specifically, ComBat removes the estimated location and scale parameters for each site from the data. In contrast, HBR estimates a *separate* location, slope, and scale for each site but does not remove them from data. This allows the model to capture different effects across sites and can accommodate unmeasured sources of variance. This feature could be useful, for example, in a lifespan study where site variation is highly correlated with age (as it is usually

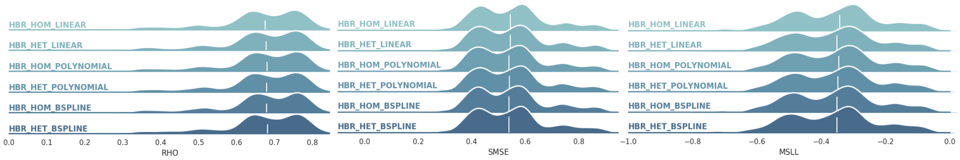

Fig S 5. Comparison between regression performance of HBRs with homoscedastic and heteroscedastic variance models. On these experimental data, using more complex heteroscedastic models provides little or no improvement over simpler homoscedastic models.

the case) and where there is heteroscedastic variance across the lifespan (*e.g.*, higher variance in older age ranges).

HBR with heteroscedastic noise model

The proposed HBR framework is capable of modeling heteroscedasticity. To evaluate the effect of accounting for heteroscedastic noise in modeling our experimental data, we conducted an experiment to compare the homoscedastic and heteroscedastic models. We have used different parametrization for parameters including linear, polynomial, and B-spline. Fig S5 summarizes the results. Given the characteristics of experimental data, using heteroscedastic noise models for the variance provide a little (in the linear case) or no improvement over the homoscedastic models while having higher model complexity.
